# B‐Cell Remodeling and Regulatory Phenotypes During Acute COVID‐19 Are Unaffected by Polyclonal Anti‐SARS‐CoV‐2 Antibody XAV‐19

**DOI:** 10.1002/eji.70284

**Published:** 2026-09-16

**Authors:** Nicolas Degauque, Richard Danger, Hoa Le Mai, Marion Cadoux, Thi‐Van‐Ha Nguyen, Gaëlle Tilly, François Raffi, Thomas Guimard, Karine Lacombe, Didier Laureillard, Laurent Hocqueloux, Vincent Dubee, Florence Ader, Cynthia Fourgeux, Jérémie Poschmann, Bernard Vanhove, Odile Duvaux, Benjamin Gaborit, Sophie Brouard, POLYCOR study group

**Affiliations:** ^1^ CHU Nantes Nantes Université INSERM, Center for Research in Transplantation and Translational Immunology UMR 1064 ITUN Nantes France; ^2^ Department of Infectious Diseases CIC 1413 CHU Nantes Nantes Université INSERM Nantes France; ^3^ Infectious Diseases and Emergency Department CHD Vendée La Roche sur Yon France; ^4^ Service des Maladies Infectieuses Et Tropicales Hôpital Saint‐Antoine Sorbonne Université INSERM Institut Pierre Louis D'épidémiologie Et De Santé Publique IPLESP GHU APHP Paris France; ^5^ Infectious and Tropical Diseases Department Caremeau University Hospital Nîmes France; ^6^ Infectious Diseases Department CHU Orléans Orléans France; ^7^ Infectious Diseases Department CHU Angers Angers France; ^8^ Département Des Maladies Infectieuses Et Tropicales Hospices Civils de Lyon Hôpital De La Croix‐Rousse Lyon France; ^9^ INSERM CNRS ENS Lyon International Centre for Research in Infectiology INSERM U1111 CNRS UMR5308 ENS Lyon Claude Bernard Lyon 1 University Lyon France; ^10^ Xenothera Nantes France

**Keywords:** B cell, severe acute respiratory syndrome coronavirus 2 (SARS‐CoV‐2), viral infection

## Abstract

Regulatory B cells (Bregs) are emerging as pivotal modulators of immune balance, yet their dynamics during severe acute respiratory syndrome coronavirus 2 (SARS‐CoV‐2) infection and their response to passive antibody therapy remain largely unexplored. In the immunological sub‐study of the randomized, placebo‐controlled POLYCOR trial evaluating XAV‐19, a glyco‐humanized polyclonal anti‐SARS‐CoV‐2 antibody, we performed longitudinal immune profiling of hospitalized COVID‐19 patients using spectral flow cytometry, in vitro functional assays, and single‐cell RNA sequencing. SARS‐CoV‐2 infection triggered a transient remodeling of the B‐cell compartment, with expansion of activated B cells and plasmablasts, contraction of switched memory B cells, and enrichment of B cells expressing regulatory‐associated markers, including PD‐L1, CD9, granzyme B, and IL‐10. Transcriptional analyses revealed dynamic reprogramming, characterized by downregulation of adhesion and costimulatory genes (CD27, ITGB7) and emergence of an interferon‐driven memory B‐cell signature (SOCS1, IFI27). In contrast, XAV‐19 treatment preserved B‐cell homeostasis, showing minimal impact on phenotype, function, and gene expression. Together, these findings uncover a transient regulatory adaptation of B cells during acute COVID‐19 and demonstrate that XAV‐19 therapy maintains immune equilibrium, reinforcing its safety and supporting the concept of immunologically neutral passive antibody therapy.

## Graphical abstract

SARS‐CoV‐2 infection drives transient B‐cell remodeling toward an activated, regulatory‐like phenotype. In this randomized sub‐study, the polyclonal neutralizing antibody XAV‐19 did not detectably alter B‐cell phenotype, transcriptome, or function relative to placebo, indicating that passive polyclonal therapy preserves B‐cell homeostasis during acute COVID‐19.

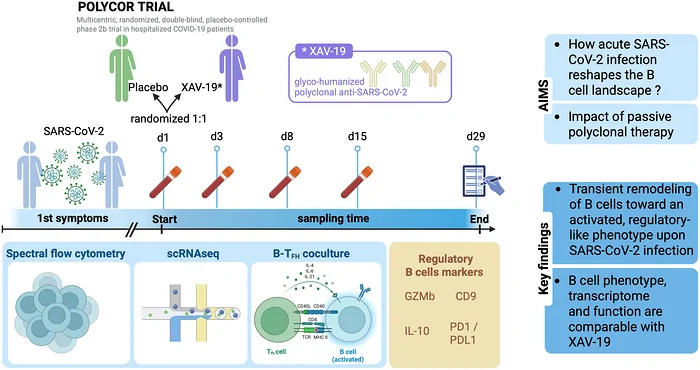

## 1 Introduction

Severe acute respiratory syndrome coronavirus 2 [SARS‐CoV‐2 (CoV‐2)] represents the third zoonotic spillover of a coronavirus into human populations within two decades. In less than 3 years, the resulting COVID‐19 pandemic has caused over 6.88 million deaths globally (Johns Hopkins University), prompting an unprecedented response. Most individuals infected with SARS‐CoV‐2 develop antibodies against surface proteins such as the spike glycoprotein and its receptor‐binding domain (RBD), with IgG titers and neutralization capacity emerging as early correlates of protection against COVID‐19 [1, 2]. However, the rapid evolution of viral variants has led to immune evasion and limited the effectiveness of neutralizing monoclonal antibodies (mAbs) [3, 4, 5, 6]. To overcome this challenge, polyclonal antibody therapies have gained attention for their ability to target multiple, non‐overlapping epitopes on the spike protein, reducing the risk of neutralization escape [7, 8, 9, 10]. XAV‐19 is a glyco‐humanized polyclonal antibody derived from genetically engineered pigs designed to minimize immunogenicity and allergic responses while enhancing complement activation and therapeutic efficacy [8, 10, 11]. Preclinical and early clinical data have supported its broad neutralizing activity across variants and favorable safety profile, making it a promising candidate, particularly in mild‐to‐moderate COVID‐19 cases not requiring oxygen therapy [7, 8]. These antibodies can provide immediate immunity, potentially accelerate recovery, and decrease severe complications by halting disease progression and reducing COVID‐19‐associated injuries such as lung damage and acute kidney injury.

The POLYCOR trial—a multicenter, randomized, double‐blind, placebo‐controlled phase 2b study—was launched in early 2021 during the transition from alpha to beta variants and enrolled 398 patients across 34 sites in France [7]. Although no clinical benefit was observed for XAV‐19 in reducing mortality or severe respiratory failure, the study incorporated a longitudinal biocollection, enabling detailed immune profiling of patients over time.

Regulatory B cells (Bregs) have been found to be directly exploited by respiratory syncytial virus (RSV), which infects them to dampen antiviral immunity and promote disease severity [12]. This precedent raised two linked questions for a respiratory infection treated with a passive polyclonal antibody: Whether SARS‐CoV‐2 similarly engages the Breg compartment and whether neutralizing antibody therapy, by binding and clearing viral antigen, perturbs that response. We leveraged this unique biocollection first to characterize the B‐cell remodeling induced by SARS‐CoV‐2 infection, with a particular attention to cells bearing a regulatory‐associated phenotype, and then to test whether XAV‐19 alters this infection‐driven trajectory.

Bregs are increasingly recognized as key modulators of immune homeostasis, exerting anti‐inflammatory functions through IL‐10 and granzyme B (GZMB) secretion and interactions with T cells and dendritic cells [13]. In various contexts—including allergy, autoimmunity, and transplantation—Bregs have been shown to suppress Th1 responses, promote regulatory T cell expansion, and contribute to long‐term tolerance [14, 15, 16, 17, 18, 19, 20, 21]. For instance, Breg expressing CD9 marker and secreting IL‐10 limit allergic airway inflammation in mice [14, 15, 16] and favor the maintenance of long‐term lung transplantation [17]. Breg notably limits the Th1 responses by impairing the function of DC [19, 20, 21] and promoting the expansion of other regulatory populations [18].

Given these findings, we hypothesized that SARS‐CoV‐2 infection and/or XAV‐19 treatment may impact Breg homeostasis, with potential consequences for the antiviral immune response. To test this hypothesis, we combined single‐cell RNA sequencing (scRNA‐seq) of purified B cells, high‐dimensional spectral flow cytometry, and in vitro B‐cell differentiation assays, using the placebo‐treated patients as the reference state of infection‐induced remodeling against which any effect of XAV‐19 could be assessed. This integrative approach aimed to define how acute SARS‐CoV‐2 infection reshapes the B‐cell landscape, including its regulatory‐associated component, and to establish whether passive polyclonal antibody therapy leaves this response intact.

## 2 Results

### 2.1 SARS‐CoV‐2 Infection Is Associated With an Expansion of B Cells With a Regulatory‐Associated Phenotype

**FIGURE 1:**
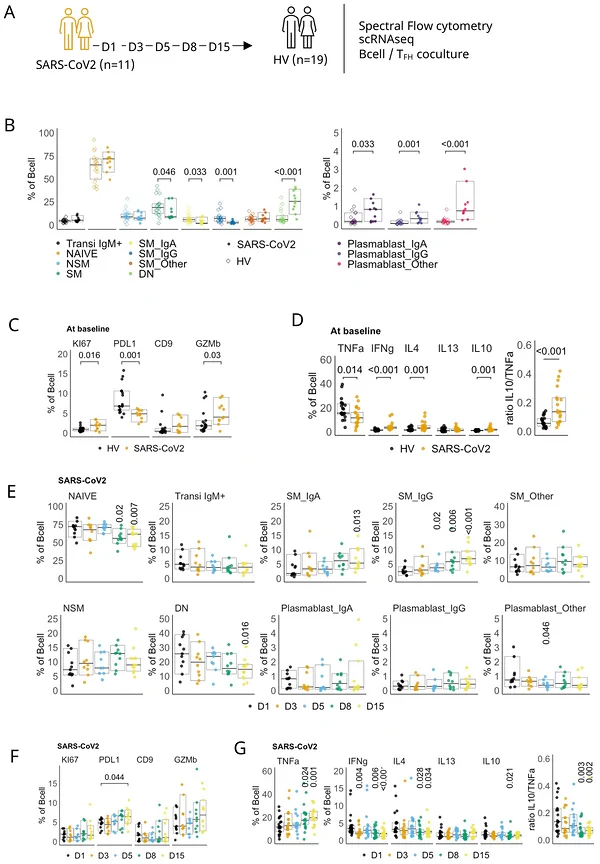
SARS‐CoV‐2 infection is associated with an expansion of B cells with regulatory‐associated phenotype. (A) Design of the study. (B–D) Phenotypic and functional characteristics of B‐cell compartment in patients at the onset of SARS‐CoV‐2 infection (*n* = 11) and in HV (*n* = 19). (E–G) Longitudinal monitoring of B‐cell compartment using phenotypic and functional markers in patients with SARS‐CoV‐2 infection. Each point represents one individual, and the box and whisker the median ± IQR. Non‐parametric test was used to compute the statistical differences, and the exact *p* value is shown. SARS‐CoV‐2, severe acute respiratory syndrome coronavirus 2; SM, switched memory.

To assess the impact of SARS‐CoV‐2 infection on the B‐cell compartment, we longitudinally analyzed peripheral blood from infected individuals (*n* = 11) collected at Days 1, 3, 5, 8, and 15 after enrollment and compared them to healthy volunteers (*n* = 19) (Figure 1A, Table S2, Figure S1). At early stages of infection, SARS‐CoV‐2 patients displayed a decrease in switched memory (SM) B cells, including CD27^+^IgD^−^IgA^+^, CD27^+^IgD^−^IgG^+^, and CD27^+^IgD^−^IgA^−^IgG^−^ subsets. Conversely, there was a significant increase in double‐negative (CD27^−^IgD^−^) B cells and plasmablasts (CD24^−^CD38^high^), particularly among IgA^+^ and IgG^+^ subsets (Figure 1B). This activation of the B‐cell compartment was further supported by an increased frequency of proliferating (Ki67^+^) B cells and elevated expression of molecules associated with regulatory potential, including PD‐L1 [22], CD9 [14, 17], and granzyme B (GZMB) [23, 24, 25, 26] (Figure 1C). Additionally, the ratio of IL‐10^+^ to TNF‐α^+^ cytokine‐secreting B cells was significantly elevated [13, 27, 28] (Figure 1D). Together, these findings indicate that acute SARS‐CoV‐2 infection promotes the expansion of activated B cells that also express phenotypic markers associated with immunoregulatory functions. We next examined the longitudinal dynamics of these changes over the course of infection (D3–D15). A progressive increase in SM IgA^+^ and SM IgG^+^ B cells was observed, accompanied by a decline in double‐negative B cells (Figure 1E). The frequency of B cells expressing regulatory‐associated markers continued to rise, with a significant increase in PD‐L1^+^ B cells and a trend toward higher frequencies of CD9^+^ and GZMB^+^ B cells (Figure 1F). In contrast, the proportion of IL‐10^+^/TNF‐α^+^ cytokine‐secreting B cells gradually decreased over time (Figure 1G), suggesting a temporal modulation of the B‐cell regulatory profile during infection.

### 2.2 SARS‐CoV‐2 Infection Induces Major Transcriptomic Changes in B Cells

**FIGURE 2:**
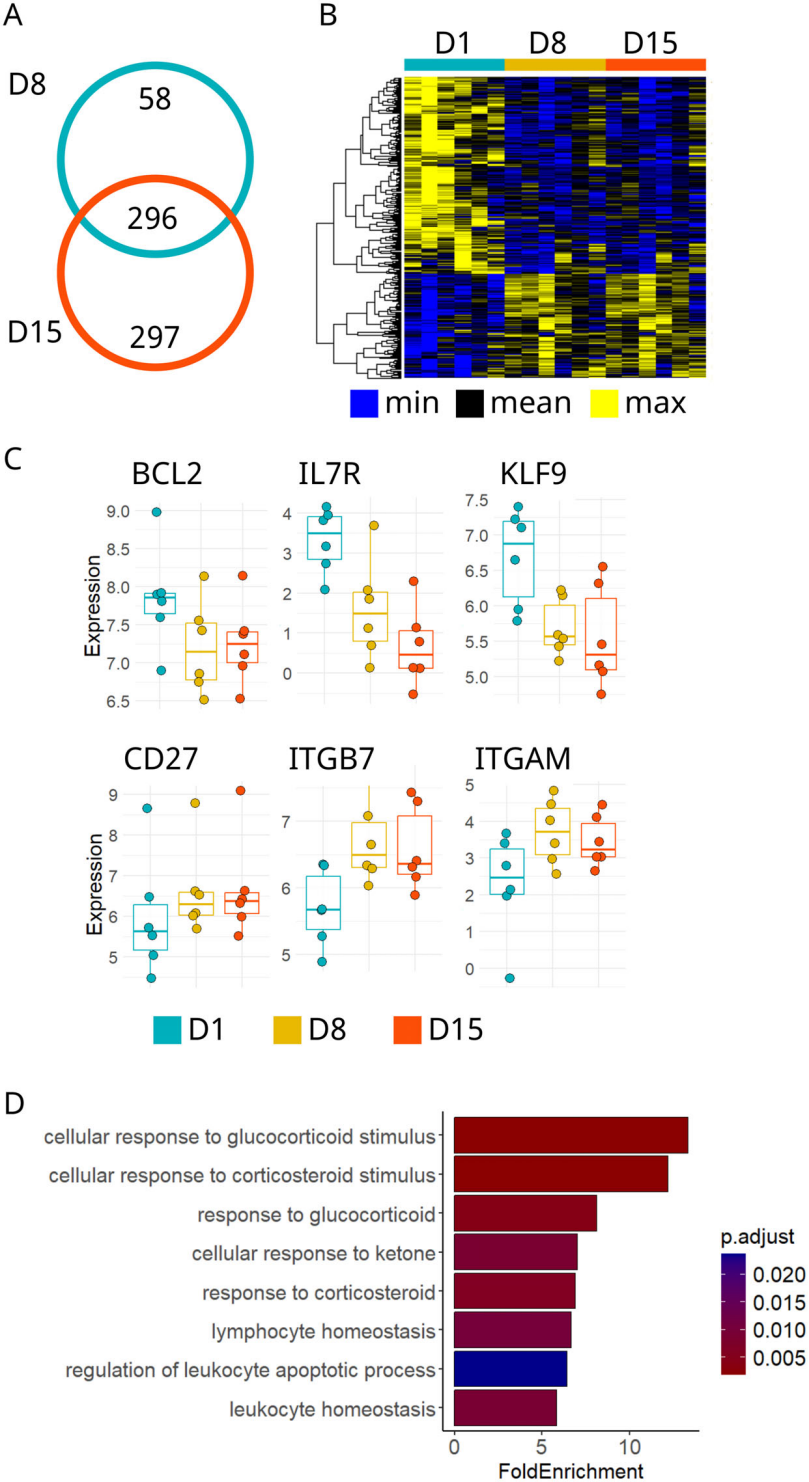
SARS‐CoV‐2 infection induces major transcriptomic changes in B cells. scRNA‐seq on enriched B cells from patients at diagnosis (Day 1, D1), and at Days 8 (D8) and 15 (D15) post‐diagnosis from placebo‐treated (*n* = 6) individuals. (A) Venn diagram displaying the overlap of differential genes at D8 and D15 compared to D1. (B–C) The expression of the 296 genes differentially expressed at D1 compared to both D8 and D15 is represented in the heatmap, and some genes are individually represented in (C). In the heatmap, blue and yellow colors represent low and high gene expression, respectively. (D) First enriched GOs within the 194 upregulated at both D8 and D15 compared to D1.

To explore how SARS‐CoV‐2 infection shapes the transcriptomic landscape of B cell, we performed scRNA‐seq on purified B cells from patients (*n* = 6) at diagnosis (Day 1, D1), and at Days 8 (D8) and 15 (D15) post‐diagnosis (Table S3). After quality control and exclusion of contaminant cells, a total of 16,158 high‐quality B cells were retained for downstream analysis (Figure S2). Compared to D1, we identified 354 and 593 differentially expressed genes (DEGs) at D8 and D15, respectively, whereas no significant differences were observed between D8 and D15 (Figure 2A,B; Figure S3). Among the 296 DEGs altered at D1 relative to both later time points, 194 were upregulated and were associated with pathways related to lymphocyte homeostasis (e.g., *IL7R*, *BCL2*) and corticosteroid response (e.g., *KLF9*), potentially reflecting treatment‐related effects during early COVID‐19 care (Figure 2C,D). Conversely, 102 genes were downregulated, including those involved in cell–cell adhesion such as *CD27*, *ITGB7*, and *ITGAM* (Figure 2C).

**FIGURE 3:**
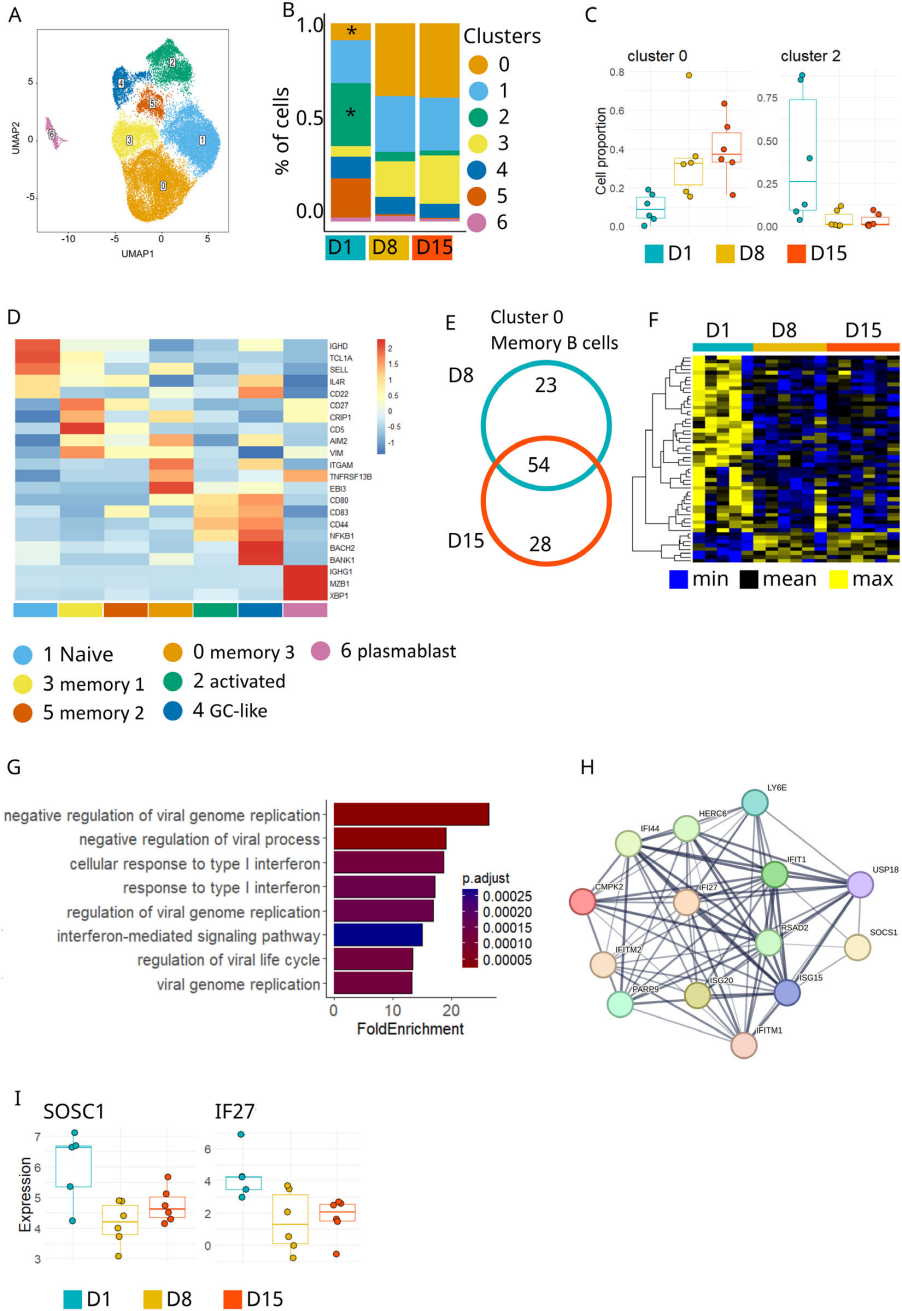
SARS‐CoV‐2 infection induces transcriptomic changes in two B‐cell clusters. (A) Uniform manifold approximation and projection (UMAP) visualization of seven B‐cell clusters identified across all time points. Each cluster is represented by a unique color and number. (B) Relative frequency of B‐cell clusters for placebo‐treated patients at different time points (D1, D8, and D15). (C) Box plots of relative frequencies for Clusters 0 and 2, which were significantly different between D1 and D8/D15 (FDR = 0.0181 and 0.0272, respectively). (D) Heatmap of gene markers for each B‐cell cluster. (E) Venn diagram showing the overlap of DEGs in Cluster 0 when comparing D1 to D8 and D15. (F) Heatmap of 54 downregulated genes in Cluster 0 at D8 and D15 relative to D1. Blue and yellow represent low and high gene expression, respectively. (G) GO enrichment analysis showing the top enriched biological processes among the 54 downregulated genes and (H) STRING protein–protein interaction network for the 54 downregulated genes. (I) Boxplots of *SOCS1* and *IFI27* expression within Cluster 0.

Clustering analysis identified seven transcriptionally distinct B‐cell clusters (Figure 3A) annotated based on canonical gene markers. Although cluster proportions varied at D1, they became more homogeneous at D8 and D15 (Figure 3B). Two clusters displayed notable temporal shifts. Cluster 0, enriched at D8 and D15 compared to D1, was associated with memory B cells, characterized by expression of *CD27*, *CRIP1*, *AIM2*, *EBI3*, *TNFRSF13B* (*TACI*), and *VIM*, consistent with the global reduction in CD27 expression observed in early infection (Figure 3C,D; Figure S2). In contrast, Cluster 2 was enriched at D01 and exhibited markers of B‐cell activation, with *CD44*, *CD80*, *CD83*, and *NFKBIA* expression (Figure 3C,D). However, no significant transcriptional differences were observed in Cluster 2 between D1 and later timepoints, suggesting a stable activation profile (Figure 3D).

In‐depth analysis of Cluster 0 (memory B cells) revealed a set of 54 downregulated genes at D8 and D15 relative to D1 (Figure 3E,F). Gene ontology (GO) enrichment identified pathways related to cellular responses to viral infection (Figure 3G). STRING network analysis further revealed a highly interconnected gene module enriched in interferon‐stimulated genes and regulators of immune responses, including *SOCS1* and *IFI27* (Figure 3H,I). These data suggest that memory B cells undergo a coordinated transcriptomic adaptation during acute SARS‐CoV‐2 infection, potentially reflecting a transient interferon‐driven state early after diagnosis.

### 2.3 XAV19 Therapy Does Not Significantly Alter the B‐Cell Compartment in SARS‐CoV‐2 Infected Patients

**FIGURE 4:**
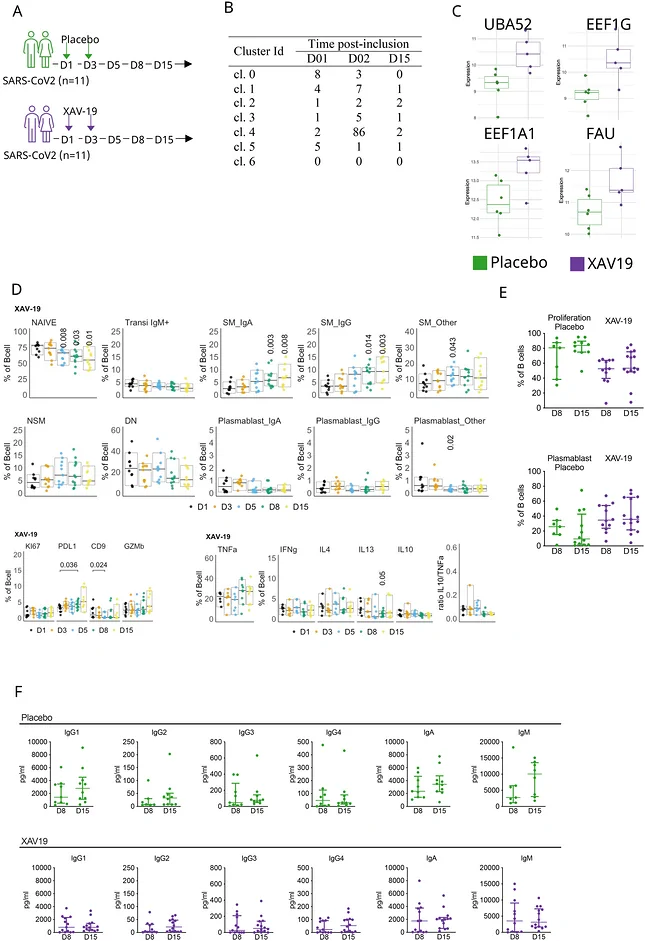
XAV19 treatment does not significantly impact the B‐cell compartment. (A) Design of the study. (B) Number of significant differentially expressed genes (DEGs) in the B‐cell clusters comparing of XAV‐19‐ to placebo‐treated patients at the three analyzed time points (D1, D8, and D15). (C) Boxplots of *UBA52*, *EEF1G*, *EEF1A1*, and *FAU* expression within Cluster 4 at D8. (D) Longitudinal monitoring of B‐cell compartment using phenotypic and functional markers in patients with SARS‐CoV‐2 infection and treated with XAV‐19. Non‐parametric test was used to compute the statistical differences, and the exact *p* value is shown. (E) Frequency of proliferating B cells and of plasmablasts after 7 days of B and Tfh coculture in placebo and the XAV‐19‐treated groups recruited at Days 8 and 15 post‐inclusion. (F) Immunoglobulins in the supernatants of B and Tfh coculture compared between Days 8 and 15 for the placebo and the XAV‐19‐treated groups. Each point represents one individual, and the box and whisker represent the median ± IQR. SM, switched memory.

We next assessed whether treatment with XAV‐19, a purified glyco‐humanized polyclonal IgG targeting multiple epitopes on the SARS‐CoV‐2 spike protein, impacted the B‐cell compartment (Figure 4A). Infusions of XAV‐19 have been shown to be safe, with a neutralizing effect demonstrated by a decrease in nasopharyngeal viral load [9]. To evaluate transcriptomic changes, we performed longitudinal scRNA‐seq analysis on enriched B cells from patients treated with XAV‐19 (*n* = 5; 12,535 cells) or placebo (*n* = 6; 16,158 cells) at diagnosis (D1) and at Days 8 (D8) and 15 (D15) post‐diagnosis (Figure S4). Overall, B‐cell transcriptomes remained highly stable across treatment groups, and no DEGs were detected in total B cells at any of the time points. Cluster‐level analysis identified only minor differences, mostly involving immunoglobulin lambda and kappa variable region genes (IGLV/IGKV) (Figure 4B). The most notable difference was observed in Cluster 4 at D8, where 86 DEGs were identified between XAV‐19– and placebo‐treated patients. This cluster was transcriptionally aligned with germinal center–like B cells and expressed *BACH2* and *BANK1* (Figure 3D). In XAV‐19–treated individuals, B cells in this cluster showed upregulation of genes encoding ribosomal proteins and translation machinery components (e.g., *EEF1A1*, *EEF1G*, *FAU*, *UBA52*), previously associated with antiviral responses [29]. Additionally, genes such as *CXCR4*, implicated in B‐cell activation during SARS‐CoV‐2 infection [30], were upregulated (Figure 4C), suggesting enhanced translational activity. These findings suggest limited and context‐specific transcriptional effects of XAV‐19 in a subset of activated B cells.

To determine whether these transcriptomic differences translated into phenotypic or functional changes, we next assessed peripheral B‐cell subsets by flow cytometry at Days 3, 5, 8, and 15 post‐diagnosis and compared them to baseline (D1, *n* = 11) (Figure 4D). B‐cell subset dynamics were similar between XAV‐19– and placebo‐treated groups. As previously observed in placebo‐treated patients, naïve B cells decreased over time, whereas SM IgA^+^ and IgG^+^ B cells expanded at later time points (Figure 1E,D). The expression of activation markers and cytokine production profiles remained largely unchanged throughout follow‐up (Figure 4D).

To address whether B‐cell features were associated with disease severity, we first compared patients admitted to the ICU by Day 29 (*n* = 3) with those who were not (*n* = 8). Principal component analysis of patient‐level parameter medians showed no separation by severity (PC1, *p* = 0.63; Figure S5). At the patient level, no parameter differed significantly between groups; the largest effect sizes, plasmablast subsets (PB IgG, PB IgA), trending higher in ICU, did not reach significance (*p* = 0.13 and 0.28; Figure S5B). A complementary timepoint‐by‐timepoint analysis was equally unrevealing, with none of 92 tests reaching significance (Figure S5C,D). Although the small number of ICU events limits statistical power and precludes formal regression modeling, these effect‐size estimates reveal no robust or consistent association between B‐cell phenotype/function and severity, in line with the parent POLYCOR trial, which evaluated clinical outcomes in the full cohort. To further probe whether B‐cell features tracked with clinical outcome, we correlated each B‐cell parameter with time to hospital discharge (length of stay) within each treatment arm. None of the B‐cell‐related parameters were statistically correlated with time to hospital discharge (median |*ρ*| = 0.26 in placebo and 0.23 in XAV‐19; Figure S6A,B). Thus, neither infection‐associated B‐cell remodeling nor XAV‐19 treatment produced a B‐cell signature associated with time to discharge. Finally, to address whether the timing of XAV‐19 administration influenced B‐cell remodeling, we first examined the distribution of treatment delay across the XAV‐19 arm. All patients were enrolled between 6 and 13 days after symptom onset (median 10 days), with no early‐treated group available for comparison (Figure S7A). Within this narrow window, we then correlated the delay from symptom onset to inclusion with each B‐cell parameter. Delay correlated weakly with B‐cell parameters in both groups with no significant correlation (Figure S7B,C). Thus, within the range of treatment timing captured by the trial, the delay to XAV‐19 administration was not associated with the magnitude of B‐cell remodeling.

Because B‐cell responses in COVID‐19 are reported to be sex‐biased [31] and our cohort was male‐predominant (17/22, 77%), we asked whether sex composition could confound the treatment comparison (Figure S8). We first included sex as a covariate in a mixed model, and none of the B‐cell parameters were significant, indicating that no treatment effect was masked by the sex imbalance. This was confirmed by the direct sensitivity analysis, showing that the XAV‐19–versus–placebo effect sizes (Cliff's delta) were highly concordant whether estimated in the whole cohort or in the male‐only subset (*r* = 0.89 across 115 parameter × timepoint comparisons), with paired differences centered on zero (Figure S8A,B). Finally, the female subgroup (3 placebo, 2 XAV‐19) was too small for a stratified female‐only comparison (minimum attainable *p* = 0.20) and is shown descriptively (Figure S8C–E). Together, these analyses indicate that the B‐cell remodeling we described and the immunological neutrality of XAV‐19 are robust to cohort sex composition, whereas acknowledging that the study is not powered to resolve sex‐specific effects.

Given that surface phenotype may not fully capture B‐cell function, we further evaluated B‐cell proliferation and plasmablast differentiation in vitro after 7 days of coculture of Tfh and B cell (Figure S9, Table S4). No significant differences were observed between XAV‐19– and placebo‐treated groups in B‐cell proliferation, plasmablast differentiation, or total immunoglobulin secretion (IgG1, IgG2, IgG3, IgG4, IgA, IgM) (Figure 4E,F). Isotype distribution also remained comparable between XAV‐19– and placebo‐treated patients. Taken together, these multi‐level analyses suggest that XAV‐19 therapy exerts limited influence on B‐cell phenotype, transcriptional profile, or functional capacity during acute SARS‐CoV‐2 infection.

## 3 Discussion

In this study, we provide an integrated phenotypic, functional, and transcriptomic analysis of B‐cell responses during acute SARS‐CoV‐2 infection, alongside with an evaluation of the immunomodulatory effects of XAV‐19, a glyco‐humanized polyclonal anti‐SARS‐CoV‐2 antibody. Our findings confirm that SARS‐CoV‐2 infection induces a dynamic remodeling of the B‐cell compartment, characterized by the expansion of activated B cells and plasmablasts, a transient reduction in SM B cells, and the emergence at the cellular level of B‐cell subsets expressing markers associated with regulatory function, including granzyme B, PD‐L1, and IL‐10.

At the transcriptomic level, SARS‐CoV‐2 infection was associated with temporally regulated changes in gene expression. These included early downregulation of adhesion and costimulatory molecules (*CD27, ITGB7, ITGAM*) and upregulation of genes involved in lymphocyte survival and corticosteroid response (*IL7R, BCL2, KLF9*), likely reflecting both viral effects and treatment‐induced modulation. Of note, nearly all patients had received corticosteroids prior to enrollment, typically 1 day before sampling. Single‐cell clustering revealed early enrichment of activated B‐cell states and a later predominance of memory B‐cell clusters. Notably, we identified a memory B‐cell–associated transcriptional module enriched in antiviral response genes (*SOCS1, IFI27*) that declined over time, suggesting a gradual resolution of the interferon‐driven state characteristic of early infection.

Beyond characterizing the temporal remodeling of B cells during acute SARS‐CoV‐2 infection, our data offer insight into the clinical immunomodulatory profile of XAV‐19. Despite being a potent polyclonal neutralizing antibody [7, 9] with neutralizing antibody levels rising significantly after infusion and an additive neutralizing effect detectable 2 to 5 days earlier than in the placebo [7]. XAV‐19 did not substantially alter B‐cell subset distribution, activation status, cytokine production, proliferation, or immunoglobulin secretion compared to placebo. Transcriptomic analyses revealed only modest changes confined to a germinal center–like cluster at Day 8, where treated patients showed increased expression of ribosomal genes and selected B‐cell–associated transcripts such as *CXCR4* and *BANK1*. These findings suggest that XAV‐19 does not substantially alter the physiological course of B‐cell responses during infection. These results are consistent with the primary clinical endpoint of the POLYCOR trial, which did not demonstrate a significant difference in disease progression between XAV‐19– and placebo‐treated patients as assessed by stay in ICU at Day 29 or time to hospital discharge (Figures S5 and S6), nor any difference between groups in the change from baseline in SARS‐CoV‐2 viral load, including when patients were stratified by the delay between symptom onset and inclusion [7] (Figure S7). The absence of B‐cell modulation reported here therefore cannot be attributed to insufficient antibody exposure and is more consistent with genuine immunological neutrality toward the B‐cell compartment. Because the immunological study is nested within this randomized, placebo‐controlled trial, the transient B‐cell dynamics driven by the infection itself are present in both arms; our conclusion of immunological neutrality is therefore a between‐arm comparison at matched timepoints, interpreted against, rather than confounded by, the natural transient course of infection.

Our findings align with prior reports that describe profound but heterogeneous B‐cell perturbations during COVID‐19, particularly in moderate to severe cases. Several studies have reported early expansion of extrafollicular B cells, plasmablasts, and double‐negative B cells—features associated with both acute antiviral defense and, in some cases, immune dysregulation [32, 33, 34]. The dynamic shift we observed from activated to memory B‐cell states over time is consistent with longitudinal analyses in COVID‐19 convalescence, which describe the gradual restoration of classical memory subsets following resolution of inflammation [35, 36]. Importantly, we identified a transient antiviral transcriptional signature within memory B cells, enriched in interferon‐stimulated genes such as *IFI27* and regulatory mediators like *SOCS1*. Similar interferon‐related programs have been reported in B cells during viral infection and may reflect early environmental imprinting by systemic cytokines [37, 38]. Consistent with a recent report that B‐cell dysregulation in acute COVID‐19 is largely transient [36], the alterations we observed, switched‐memory contraction, double‐negative expansion, and the elevated IL‐10/TNF‐α ratio, progressively resolved across follow‐up, as did the interferon‐stimulated memory signature (SOCS1, IFI27).

A notable aspect of our study is the emergence of B cells expressing phenotypic features associated with regulatory function, including PD‐L1, CD9, GZMB, and IL‐10 during SARS‐CoV‐2 infection. Each of these markers has documented Breg associations in humans, including granzyme B– and CD9–expressing Breg subsets that we and others have previously characterized in autoimmune, transplant, and viral settings [14, 17, 23, 24, 25, 26] and PD‐L1hi Bregs that suppress follicular helper T cell–dependent humoral responses [22]. Although these cells were not functionally validated as bona fide regulatory B cells (Bregs), their phenotype is reminiscent of IL‐10–producing Bregs described in other infectious and inflammatory contexts [13, 27, 28]. In RSV infection, Bregs were shown to be directly infected and subverted by the virus to dampen antiviral immunity and promote viral persistence [12]. Although we did not observe evidence of viral hijacking, the early expansion of IL‐10^+^ B cells during SARS‐CoV‐2 infection may represent a counter‐regulatory response to inflammation. Whether these cells are beneficial, pathogenic, or functionally inert in this context remains to be determined. Despite its strengths, our study has several limitations. First, the sample size—particularly for scRNA‐seq and functional assays—was limited due to logistical constraints during the pandemic. Although our findings are consistent across multiple levels of analysis, larger cohorts are needed to confirm the generalizability of the observed B‐cell dynamics and transcriptional programs. Second, our phenotypic characterization of regulatory B cells was based on surrogate markers and cytokine expression, without definitive functional validation of suppressive capacity in vivo; we therefore describe a regulatory‐associated phenotype rather than functionally defined Bregs, and exhausted biobanked material precluded the suppression analyses that would resolve this. Third, we focused exclusively on circulating B cells; tissue‐resident populations, including those in the lung and lymphoid organs, may exhibit distinct responses that remain unexplored. SARS‐CoV‐2‐specific memory T and B cells with tissue‐resident profiles were identified in lung and lung‐associated lymph nodes that likely contribute to site‐specific protective immunity against future infections [39, 40]. Because our analysis focused on the global B‐cell compartment, we cannot exclude that XAV‐19 modulates the endogenous virus‐specific (spike/RBD) humoral response. Profiling of SARS‐CoV‐2–specific B cells and germinal‐center–focused analyses would be required to address this, restricting our conclusions to the peripheral, global B‐cell compartment. Finally, the lack of impact observed for XAV‐19 on B cells does not exclude potential effects on other immune compartments, such as monocytes or T cells, which warrant future investigation. In addition, longitudinal sampling ended at D15, capturing the acute‐phase remodeling and its early resolution but not full normalization to a pre‐infection baseline; this bounds the temporal scope of our observations without affecting the between‐arm comparison.

In conclusion, our data provide a comprehensive multi‐modal profile of B‐cell responses during acute SARS‐CoV‐2 infection and suggest that XAV‐19 therapy does not significantly alter B‐cell immunity. These findings support the immunological neutrality of XAV‐19 in terms of B‐cell modulation and underscore the need for deeper functional analyses of regulatory B‐cell subsets in the context of viral infection and antibody‐based immunotherapy.

## 4 Methods

### 4.1 Clinical Data

This study was conducted within the framework of the multicenter, randomized, double‐blind, placebo‐controlled clinical trial POLYCOR, previously published [7, 8, 9]. Adults aged 18–85 years were eligible if they had a confirmed diagnosis of SARS‐CoV‐2 infection by reverse transcription PCR (RT‐PCR) within 14 days of symptom onset and required hospitalization for low‐flow oxygen therapy (oxygen saturation ≥92% with oxygen supplementation ≤6 L/min). The trial was performed in accordance with Good Clinical Practice guidelines, applicable regulatory requirements, and the principles of the Declaration of Helsinki. The study protocol was approved by the Ethics Committee OUEST VI (approval number 20.06.15.31306), and written informed consent was obtained from all participants before enrollment. The trial is registered at ClinicalTrials.gov (identifier NCT04453384). An ancillary immunological study enrolled 37 patients (16 in the placebo group and 21 in the XAV‐19 group), with blood samples collected at Days 1, 3, 5, 8, and 15 post‐diagnosis and stored for subsequent analyses. Clinical characteristics of the patient cohort are summarized in Table 1 and in Tables S2–S4, according to the analytic readouts.

**TABLE 1:** Baseline characteristics and clinical outcomes of patients, stratified by treatment group.

Variable	Placebo (*n* = 16)	XAV‐19 (*n* = 21)	*n*	*p*
**Demographics**				
Age, years	53 [49–58] (42–75)	57 [47–62] (36–83)	16/21	0.902
Sex, female	4/16 (25%)	4/21 (19%)	16/21	0.705
BMI, kg/m^2^	28.4 [26.0–31.8] (22.5–40.0)	31.4 [27.7–34.4] (19.4–40.6)	16/21	0.334
Delay first symptom‐screening, days	10 [9–11] (4–11)	9 [6–10] (3–13)	16/21	0.208
**Baseline severity**				
SpO2 at rest, %	94 [93–95] (92–96)	93 [92–94] (90–95)	16/21	0.022
NEWS score	5 [4–8] (3–8)	7 [5–10] (3–12)	15/21	0.051
PaO2/FiO2 at D1	254 [215–287] (172–358)	232 [182–261] (157–400)	16/21	0.325
**Baseline biology**				
CRP, mg/L	74.5 [56.3–90.7] (36.3–129.6)	53.8 [30.0–112.2] (14.4–196.7)	12/10	0.346
Ferritin, µg/L	946 [506–1244] (196–1975)	896 [822–1414] (220–2435)	11/10	0.654
Viral load D1, log	5.0 [4.3–6.6] (0.0–7.2)	5.2 [3.3–5.6] (1.1–7.4)	12/9	0.917
Anti‐SARS‐CoV‐2 serology positive	8/14 (57%)	13/19 (68%)	14/19	0.716
**Co‐treatment**				
Corticosteroids at inclusion	14/16 (88%)	19/21 (90%)	16/21	1.000
Corticosteroid delay, days	−1 [−1 to 0] (−4 to 3)	−1 [−2 to 0] (−2 to 0)	16/19	0.754
**Outcome**				
Ordinal scale D8	3 [2–4] (1–4)	3 [3–4] (1–6)	16/21	0.773
Ordinal scale D15	2 [1–2] (1–3)	2 [1–2] (1–6)	16/21	0.556
PaO2/FiO2 at D8	376 [314–434] (136–457)	329 [304–457] (75–690)	16/21	0.793
PaO2/FiO2 at D15	410 [410–533] (348–762)	410 [376–457] (81–690)	16/21	0.365
Time to discharge, days	8 [7–11] (4–14)	8 [5–10] (4–37)	16/21	0.589
Viral load D8, log	1.9 [1.1–4.7] (0.0–5.4)	0.0 [0.0–3.9] (0.0–5.0)	10/9	0.178
Viral load D15, log	0.0 [0.0–1.1] (0.0–2.8)	0.0 [0.0–0.6] (0.0–3.9)	11/7	0.871
ICU admission by D29	3/16 (19%)	4/21 (19%)	16/21	1.000

*Note*: Values are median [IQR] (min–max) for continuous variables and *n*/*N* (%) for categorical variables. *p* from a two‐sided Mann–Whitney (Wilcoxon rank‐sum) test for continuous variables and Fisher's exact test for categorical variables.

Abbreviation: SARS‐CoV‐2, severe acute respiratory syndrome coronavirus 2.

### 4.2 Blood Samples

PBMCs were isolated from EDTA‐anticoagulated blood by density gradient centrifugation using Ficoll, following the manufacturer's instructions, and cryopreserved in autologous serum supplemented with 10% DMSO. PBMCs were then thawed in CTL wash supplemented with benzonase (10 U/mL).

### 4.3 Reagents

The antibodies used for flow cytometry analyses and cell sorting and key reagents are listed in Tables S5 and S6.

### 4.4 Immune Profiling by Spectral Flow Cytometry

Two million PBMCs were either directly stained with the “B‐cell panel” or stimulated with PMA (50 ng/mL) and calcium ionophore (500 ng/mL) for 3.5 h in the presence of brefeldin A (5 µg/mL), followed by staining with the “functional panel” targeting IL‐10, IL‐13, IL‐17, IFN‐γ, and TNF‐α. Cells were analyzed using a five‐laser Aurora spectral flow cytometer (Cytek). Each antibody was titrated individually, and a deconvolution matrix was established according to Cytek recommendations. Quality control procedure was performed prior to each acquisition run, following Cytek's recommendations. Unmixed FCS files were first subjected to quality control using the PeacoQC [41] algorithm. B‐cell populations were identified by sequential gating on CD45^+^Singlet^+^LiveDeadViabilityDye‐CD3^−^CD19^+^CD20^+^HLADR^+^ cells (Figure S1A). Subsets of B cells were further identified using a supervised gating (Figure S1B,C). Data analyses were performed using OMIQ (https://www.omiq.ai/).

### 4.5 Single‐Cell RNA Sequencing

Total lived B cells (CD3^−^CD19^+^FVD^−^) were isolated from PBMCs using an ARIA III sorter (BD Biosciences). Cells were labeled with sample‐specific DNA‐barcoded antibodies (HashTag Oligonucleotide, 10× genomics), according to CITE‐seq protocols, and pooled in equal proportions. A total of 20,000 cells were loaded onto a chromium controller (10× genomics) for single‐cell encapsulation, and libraries were prepared. Sequencing was performed on a Nova‐Seq 6000 (Illumina) at the GenoBird platform (IRS‐UN, CHU Nantes). Raw sequencing data underwent quality control with FastQC and were processed using the CellRanger pipeline (v3.1.0) with default parameters. FASTQ files were aligned to the GRCh38 reference genome. Downstream analysis was conducted using the R Seurat package (v.5.2.0) [42]. Demultiplexing and initial preprocessing included the exclusion of low‐quality cells defined by expression of <500 or >3000 genes or >7.5% of mitochondrial gene content. Non‐B cells were removed on the basis of the expression of lineage‐specific genes (*CD3E*, *CD4*, *CD8A* for T lymphocytes, *CD14*, and *FCGR3A* for monocytes and *PF4* for platelets) (Figure S2). Data normalization and batch effect correction were performed using SCTransform (v2). Integration across samples was conducted using Seurat's data integration workflow, excluding immunoglobulin‐, ribosomal‐, HLA‐, and mitochondrial‐related genes to avoid technical bias. Integrated datasets were clustered and annotated on canonical marker genes and results from the FindAllMarkers function (positive markers expressed in at least 20% of cells in one of the two groups, only positively expressed with a log fold change of at least 1.5). Cell‐type proportions were compared using the propeller method from the *speckle* R package [43]. Differential gene expression between experimental conditions was analyzed using a pseudobulk approach with the *limma* R package and *voom* correction [44]. Genes with total read counts <100 were excluded. GO enrichment analysis was performed using the *clusterProfiler* R package [45], and protein interaction networks were constructed using the STRING database [46]. All scRNA‐seq analyses were performed with R software version 4.2.1.

### 4.6 In Vitro B‐Cell Proliferation, Plasmablast Differentiation, and Immunoglobulin Production

After thawing, PBMCs were stained with fluorescence‐conjugated antibodies against CD3, CD4, CD19, CD45RA, and CXCR5, and B cells (CD19^+^) and follicular helper T cells (Tfh; CD3^+^CD4^+^CXCR5^+^CD45RA^−^) were sorted using an FACS Aria II cytometer (BD Biosciences; Figure S9A). Sorted B cells were labeled with a Cell Proliferation Dye (CPD eFluor 670) and cocultured with autologous Tfh cells for 7 days in 96‐well U‐bottom plates at a 1:1 ratio, in the presence of CytoStim (Miltenyi Biotec) as a polyclonal activator. On Day 7, supernatants were stored at −20°C for subsequent immunoglobulin quantification. For immunophenotyping, cells were stained with a fixable viability dye (FVD eFluor450), followed by antibodies against CD4, CD19, CD27, and CD38, and acquired on an FACS Canto II cytometer (BD Biosciences; Figure S9B). Immunoglobulin concentrations (IgG1, IgG2, IgG3, IgG4, IgA, and IgM) in culture supernatants were measured using a customized LEGENDplex 6‐plex Human Immunoglobulin Panel (BioLegend), following the manufacturer's instructions.

### 4.7 Statistical Analysis

Continuous variables were compared using the nonparametric Mann–Whitney *U* test and categorical variables by Fisher's exact test. Data are presented as medians with 95% CI. Throughout, D3 was excluded for sparse coverage, and figures were generated in GraphPad Prism v9 and R (v4.2.1; revision analyses in v4.3.3).

#### 4.7.1 Correlation With Continuous Clinical Variables

B‐cell parameters were correlated with time to hospital discharge (each arm separately) and, within the XAV‐19 arm, with symptom‐to‐inclusion delay (parameter values and change from baseline), using Spearman's *ρ* across patients at each post‐inclusion timepoint rather than a median split, given the skewed distributions. Because parameters are strongly intercorrelated, panel‐level significance was assessed by a maximum‐statistic permutation test: The observed maximum |*ρ*| across all parameter × timepoint pairs was referred to a null distribution from 2000 permutations of the clinical variable—preserving inter‐parameter structure while breaking the clinical association—with the permutation *p* value defined as the fraction of permutations reaching the observed max |*ρ*|.

#### 4.7.2 Association With Disease Severity

Patients were stratified by ICU admission at D29 (3 ICU, 8 non‐ICU), and each parameter was summarized per patient as the median across timepoints. PC1 from a PCA of scaled patient‐level medians (23 parameters) was compared between groups by Mann–Whitney; univariate effects were reported as Cliff's *δ* with exact *p* values, complemented by per‐timepoint Mann–Whitney tests. Given the low event count and exploratory intent, no multiplicity correction was applied.

#### 4.7.3 Sex Sensitivity Analysis

To assess whether sex composition confounded the treatment comparison (all five timepoints), we (i) fitted a linear mixed model per parameter, using logit‐transformed percentage or log‐transformed ratio in function of ∼timepoint + arm + sex + age + (1 | patient), by REML (lme4 R package). *p* values were corrected using Benjamini–Hochberg correction (BH). Parameters with <8 patients or singular fits excluded; and (ii) summarized XAV‐19 versus placebo at each parameter × timepoint by Cliff's *δ* (95% CI) and Hodges–Lehmann shift, with BH‐corrected Mann–Whitney *p* values, in the whole cohort and in the male‐only subset, quantifying concordance between the two sets of effect sizes. Female patients were shown as a descriptive overlay without testing.

## Author Contributions

Conceptualization and design: Nicolas Degauque, Richard Danger, Hoa Le Mai, and Sophie Brouard. Acquisition of data: Nicolas Degauque, Richard Danger, Hoa Le Mai, Marion Cadoux, Thi‐Van‐Ha Nguyen, Gaëlle Tilly, and Cynthia Fourgeux. Methodology: Jérémie Poschmann. Formal analysis and interpretation of data: Nicolas Degauque, Richard Danger, Hoa Le Mai, and Sophie Brouard. Clinical data management and patient follow‐up: François Raffi, Thomas Guimard, Karine Lacombe, Didier Laureillard, Laurent Hocqueloux, Vincent Dubee, Florence Ader, and Benjamin Gaborit. Funding acquisition: Sophie Brouard, Benjamin Gaborit, Odile Duvaux, and François Raffi. Writing – original draft: Nicolas Degauque. Writing – review and editing: Nicolas Degauque, Richard Danger, Hoa Le Mai, Benjamin Gaborit, Bernard Vanhove, François Raffi, and Sophie Brouard.

## Funding

The POLYCOR study was funded and fully supported by the University Hospital of Nantes with support from the Bpifrance (Banque Publique d'Investissement) (BPI France) in the framework of the Investment for the Future Program (Programme d'Investissements d'Avenir) and Xenothera.

## Ethics Statement

The study protocol was approved by the Ethics Committee OUEST VI (approval number 20.06.15.31306).

## Consent

Written informed consent was obtained from all participants before enrollment.

## Conflicts of Interest

B.G. reports receipt of nonfinancial support from Gilead Sciences and MSD, outside the submitted work. B.V. is an employee and chief scientific officer/operating officer of Xenothera and owns shares and holds share options in Xenothera. K.L. reports receipt of personal fees and nonfinancial support from AbbVie, Chiesi, Healthcare, Janssen, MSD, and ViiV, outside the submitted work. O.D. is a cofounder of Xenothera, is the CEO of Xenothera, and owns shares and holds share options in Xenothera. F.R. reports receipt of personal fees from AbbVie, AstraZeneca, Gilead Sciences, Janssen, Merck, Roche, ViiV Healthcare, and Xenothera, outside the submitted work. All other authors declare no conflicts of interest.

## Clinical Trial Registration

The trial is registered at ClinicalTrials.gov (identifier NCT04453384).

## Supporting information




**Supporting File**: eji70284‐sup‐0001‐SuppMat.pdf.

## Data Availability

The scRNA‐seq datasets generated during this study have been deposited in the European Nucleotide Archive (PRJEB98240).
