# Supplementary material for: B‐Cell Remodeling and Regulatory Phenotypes During Acute COVID‐19 Are Unaffected by Polyclonal Anti‐SARS‐CoV‐2 Antibody XAV‐19
Source: Eur J Immunol. 2026 Sep 16;56(9):e70284. doi: 10.1002/eji.70284 (PMC13579198; doi:10.1002/eji.70284)
Supplement: Supplementary file 1 [file EJI-56-e70284-s001.pdf]

Supplementary Figures

Supplementary Figure 1. Phenotype of B cell subsets.

Supplementary Figure 2. Single-cell analysis of B cells.

Supplementary Figure 3. Volcano plot displaying the differential analysis at D8 and D15 compared to D1 in all B cells.

Supplementary Figure 4. Volcano plot displaying the differential analysis at D8 and D15 compared to D1 within the cluster 0.

Supplementary Figure 5. Exploratory analysis of B-cell parameters by disease severity (ICU vs non-ICU at day 29).

Supplementary Figure 6. B-cell parameters do not correlate with time to hospital discharge in either treatment arm.

Supplementary Figure 7. B-cell parameters do not vary with the delay from symptom onset to XAV-19 administration.

Supplementary Figure 8. B cell remodelling and the XAV-19 treatment effect are robust to cohort sex composition.

Supplementary Figure 9. Gating strategies used to isolate B cells and follicular helper T cells (Tfh) and to assess B cell proliferation and plasmablast differentiation after coculture.

Supplementary Table

Supplementary Table 1. POLYCOR Group author's list

Supplementary Table 2. Baseline characteristics and clinical outcomes of patients included in the flow cytometry analysis, stratified by treatment group.

Supplementary Table 3. Baseline characteristics and clinical outcomes of patients included in the scRNAseq analysis, stratified by treatment group.

Supplementary Table 4. Baseline characteristics and clinical outcomes of patients included in the B/T_{FH} analysis, stratified by treatment group.

Supplementary Table 5. Listing of antibodies

Supplementary Table 6. Listing of reagents

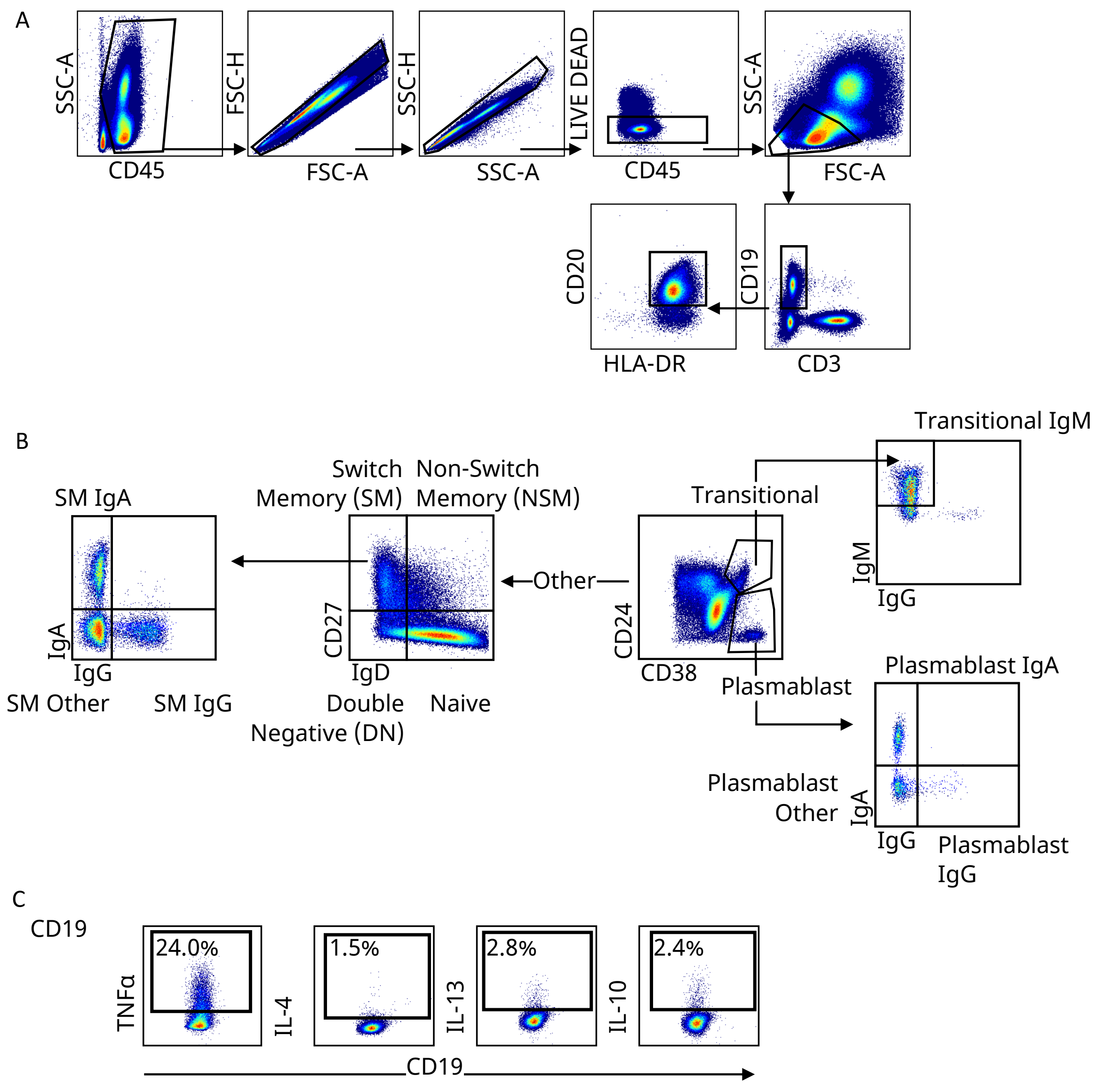

Supplementary Figure 1. Phenotype of B cell subsets. (A) Gating strategy to identify B cell. **(B–C)** Phenotype of the 8 B cell subsets and analysis of cytokine secretion upon PMA/Ionomycin restimulation. A representative example of the sequential gating strategy is shown.

A

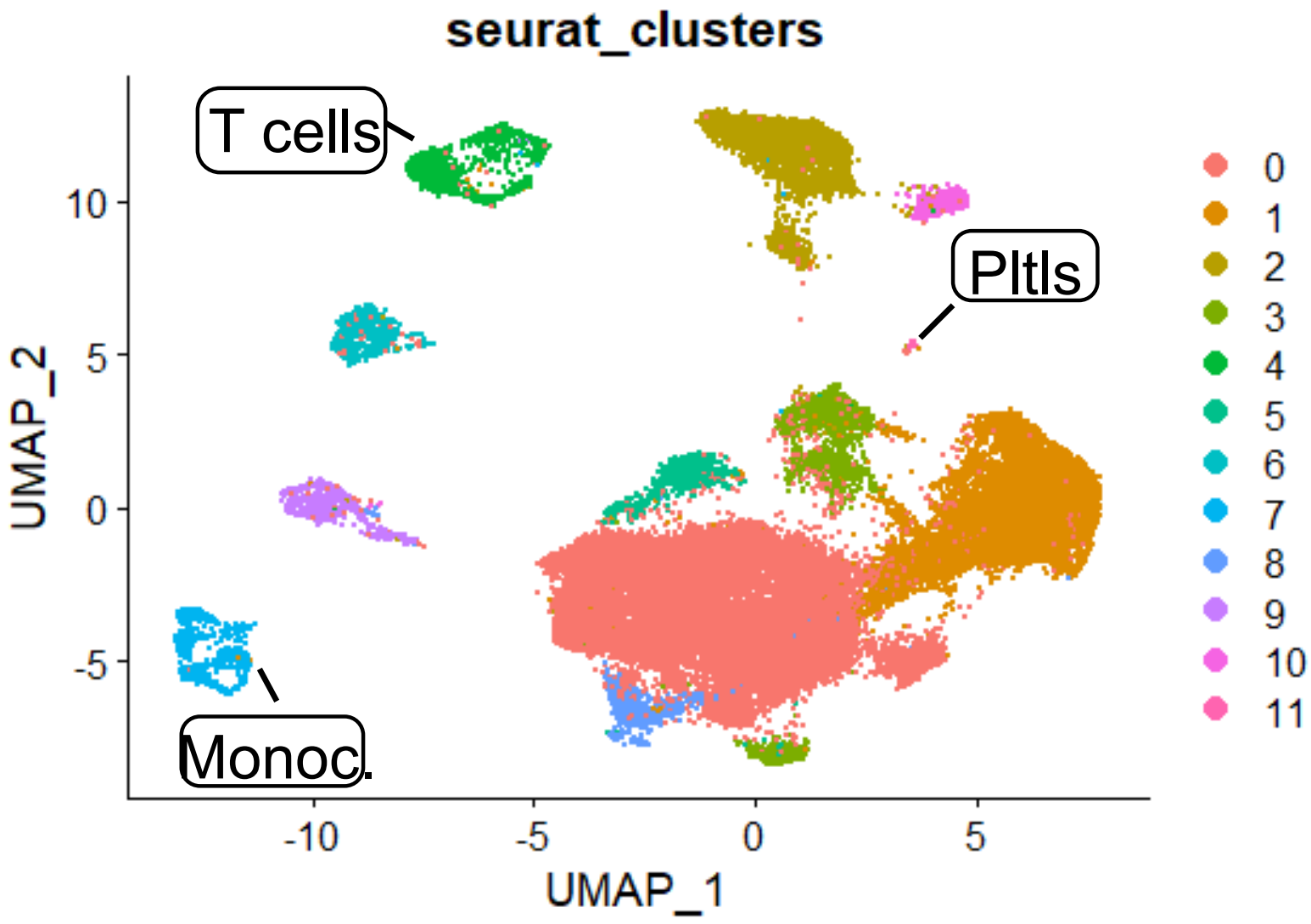
seurat_clusters
T cells
Pltls
Monoc
UMAP_2
UMAP_1
10
5
0
-5
-10
0
1
2
3
4
5
6
7
8
9
10
11

B

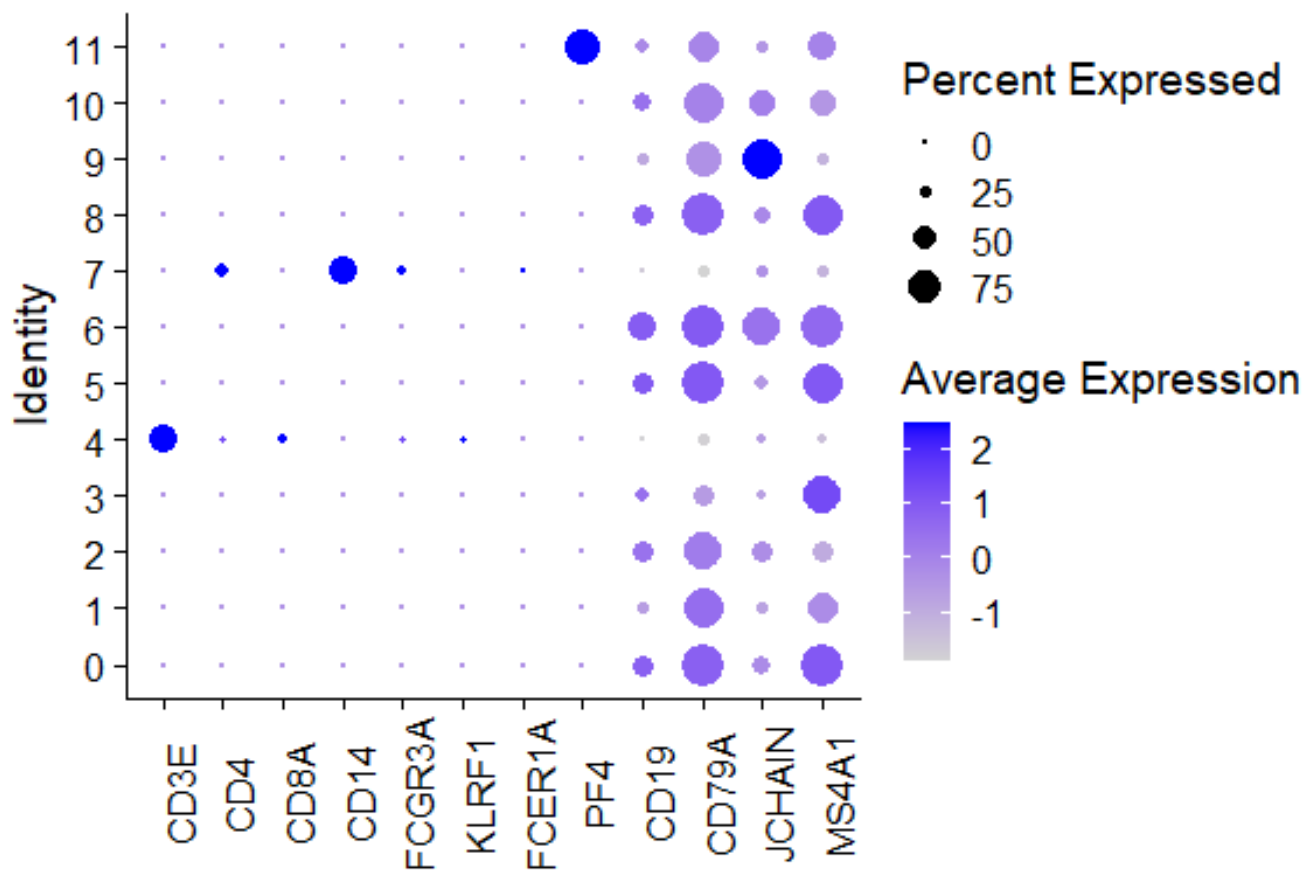
Identity
CD3E
CD4
CD8A
CD14
FCGR3A
KLRF1
FCER1A
PF4
CD19
CD79A
JCHAIN
MS4A1
Percent Expressed
0
25
50
75
Average Expression
2
1
0
-1

C

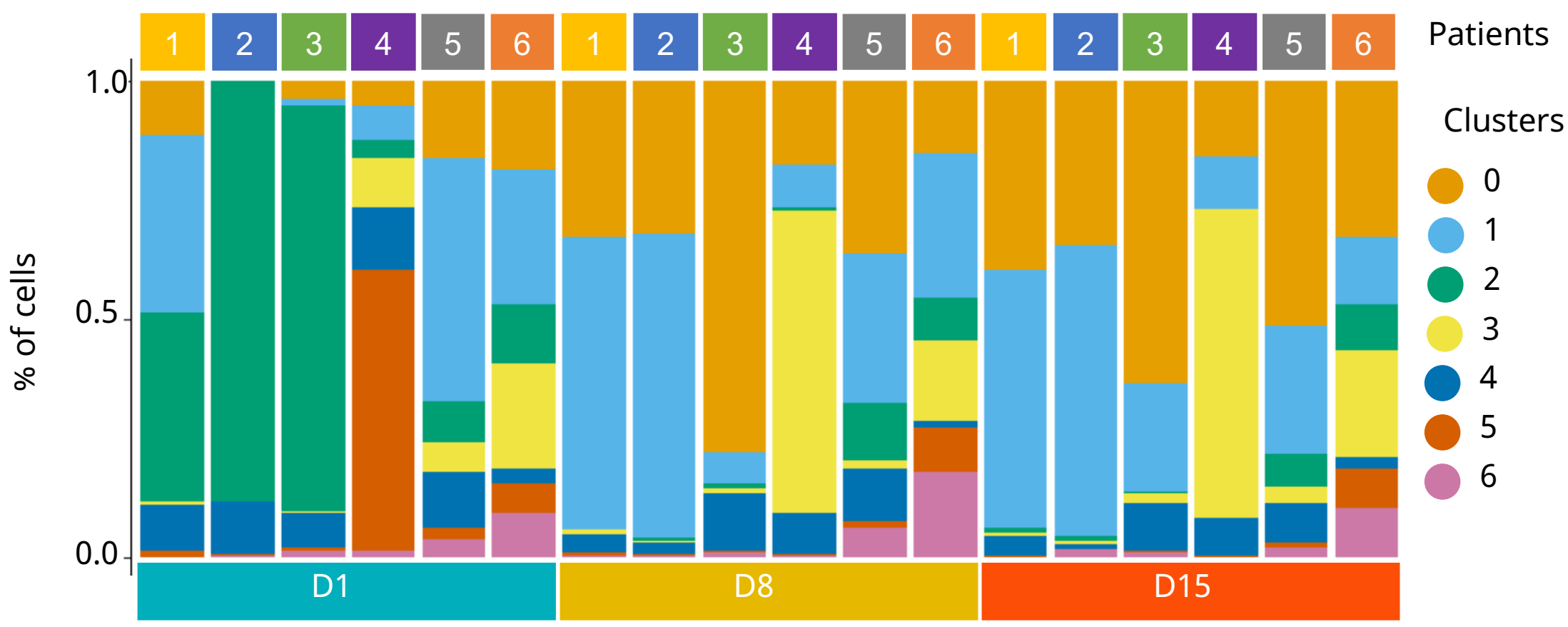
% of cells
1.0
0.5
0.0
Patients
Clusters
D1
D8
D15
1
2
3
4
5
6

Supplementary Figure 2. Single-cell analysis of B cells. (A) UMAP visualization of analyzed cells displaying contaminating non-B cells (T cells, platelets and monocytes) highlighted for exclusion. **(B)** The dot plot represents gene expression profiles of specific marker genes for major cell population. **(C)** Diagram showing the relative frequencies of clusters for each placebo-treated patient individually, at each time point.

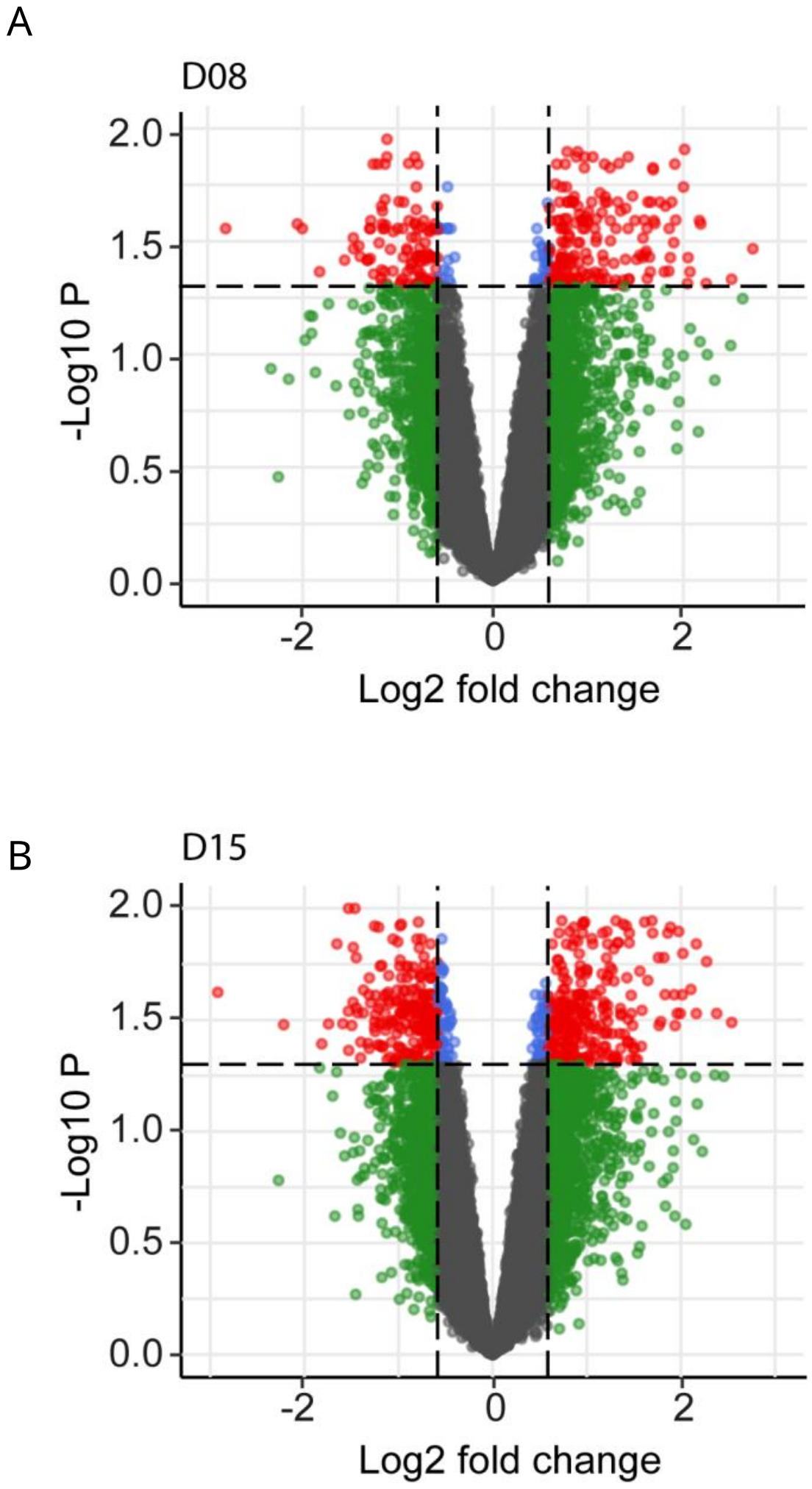

Supplementary Figure 3. Volcano plot displaying the differential analysis at D8 (A) and D15 (B) compared to D1 in all B cells. The x-axis represents the $\log_2$ fold change ($\log_2$FC), and the y-axis represents the $-\log_{10}$ adjusted p-value (FDR). DEGs are highlighted in red. Non-DEGs are shown in blue and green to represent non-significant genes.

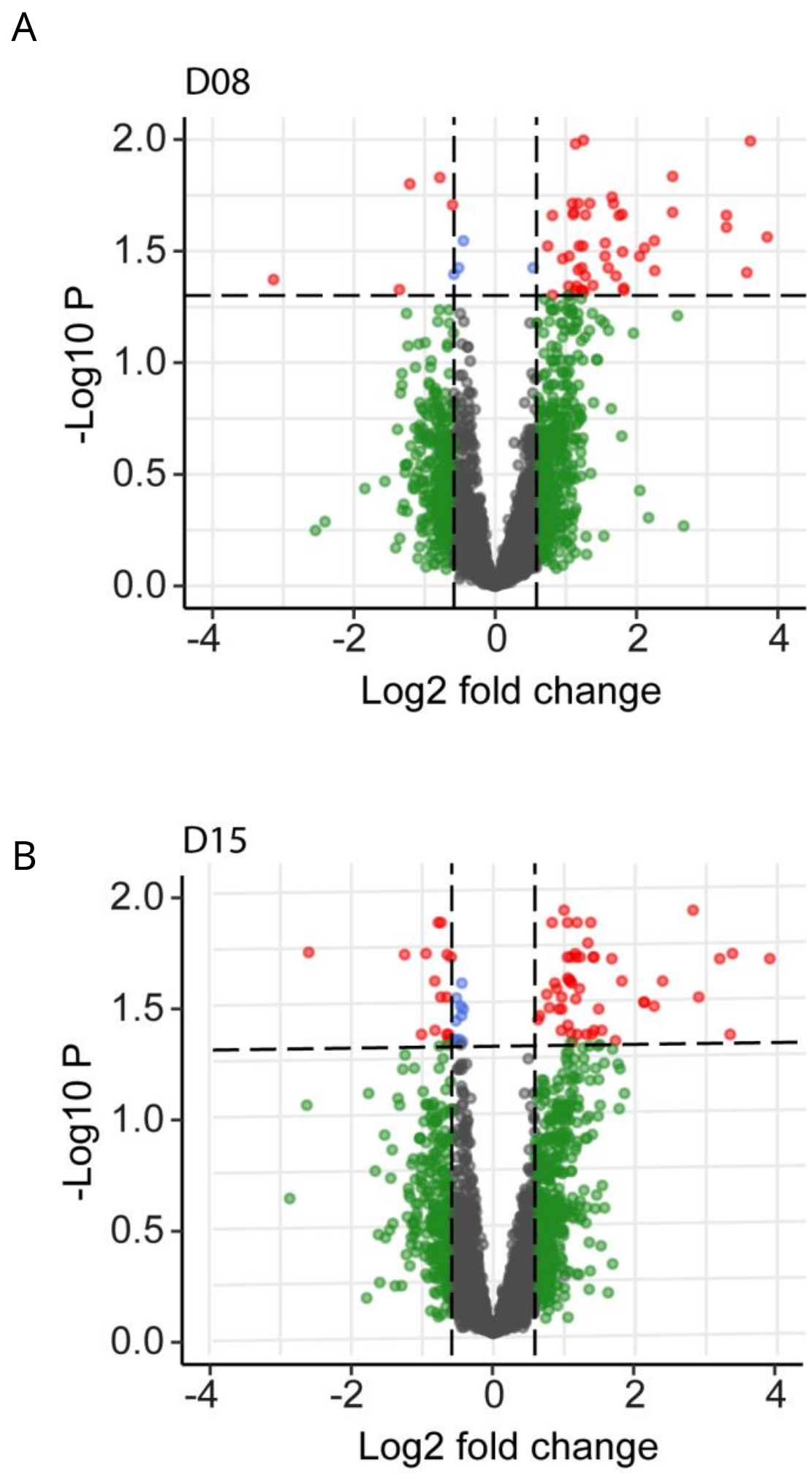

Supplementary Figure 4. Volcano plot displaying the differential analysis at D8 (A) and D15 (B) compared to D1 within the cluster 0. The x-axis represents the $\log_2$ fold change ($\log_2$FC), and the y-axis represents the -$\log_{10}$ adjusted p-value (FDR). DEGs are highlighted in red. Non-DEGs are shown in blue and green to represent non-significant genes.

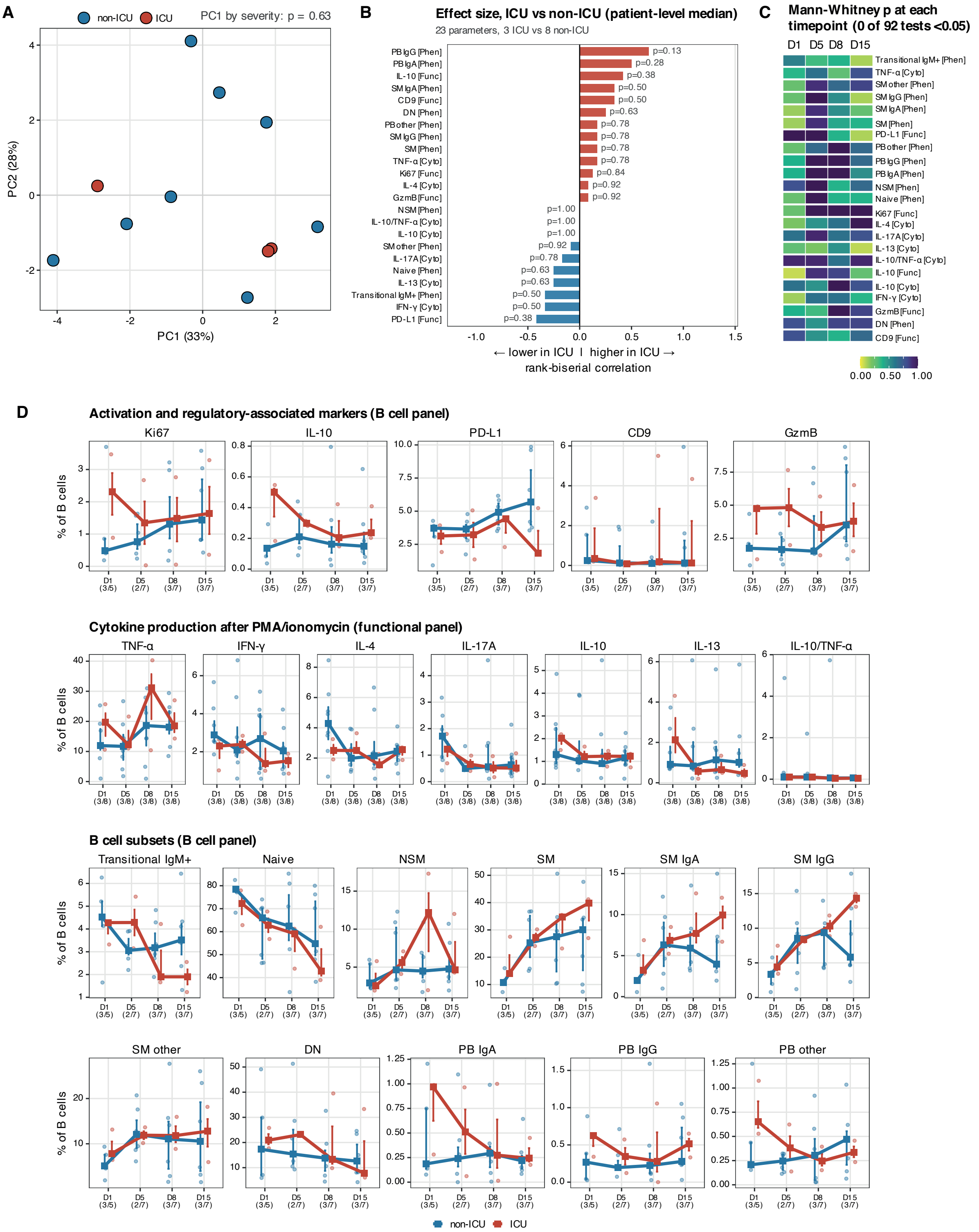

Supplementary Figure 5. Exploratory analysis of B-cell parameters by disease severity (ICU vs non-ICU at day 29). (**A**) PCA of patient-level median values across 23 B-cell parameters; each point is one patient. PC1 did not separate ICU from non-ICU patients (Mann–Whitney p = 0.63). (**B**) Effect sizes (rank-biserial correlation) for the ICU vs non-ICU

comparison of patient-level medians (23 parameters; 3 ICU vs 8 non-ICU). Bars to the right indicate higher levels in ICU, to the left lower levels in ICU; exact Mann–Whitney p-values are shown. No parameter reached significance. (**C**) Heatmap of exact Mann–Whitney p-values for each parameter at each timepoint (D1, D5, D8, D15); 0 of 92 tests reached $p < 0.05$. (**D**) Longitudinal trajectories of activation- and regulatory-associated markers (B-cell panel), cytokine production after PMA/ionomycin (functional panel), and B-cell subsets (B-cell panel) in non-ICU (blue) and ICU (red) patients. Symbols show the group median and error bars the 95% CI; sample sizes are indicated as (ICU/non-ICU) per timepoint.

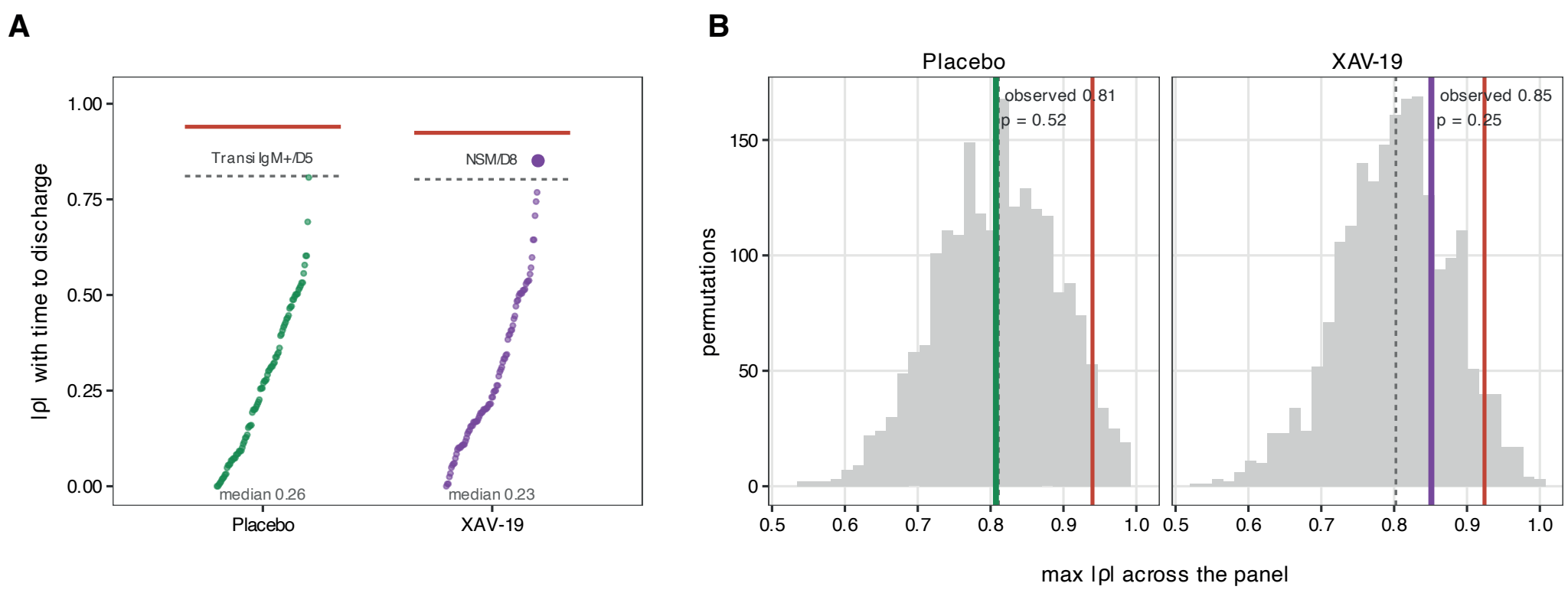

Supplementary Figure 6. B-cell parameters do not correlate with time to hospital discharge in either treatment arm. Time to discharge was correlated with each B-cell parameter at each timepoint by Spearman's ρ, separately for placebo (green) and XAV-19 (purple) patients. (**A**) Absolute Spearman correlations (|ρ|) for all parameter × timepoint pairs, ranked in ascending order within each arm; each point is one parameter at one timepoint. The strongest association per arm is labelled (placebo: Transitional IgM+ at D5; XAV-19: NSM at D8) and marked by the dashed line (observed max |ρ| = 0.81 and 0.85, respectively); the panel-wide median |ρ| is annotated. The red line indicates the permutation significance threshold; both observed maxima fall below it. (**B**) Null distribution of the panel-wide max |ρ| obtained from [N] permutations of time to discharge (grey histogram), per arm. The observed max |ρ| (coloured line) lies within the bulk of the null distribution in both arms (placebo p = 0.52; XAV-19 p = 0.25); the red line marks the significance threshold.

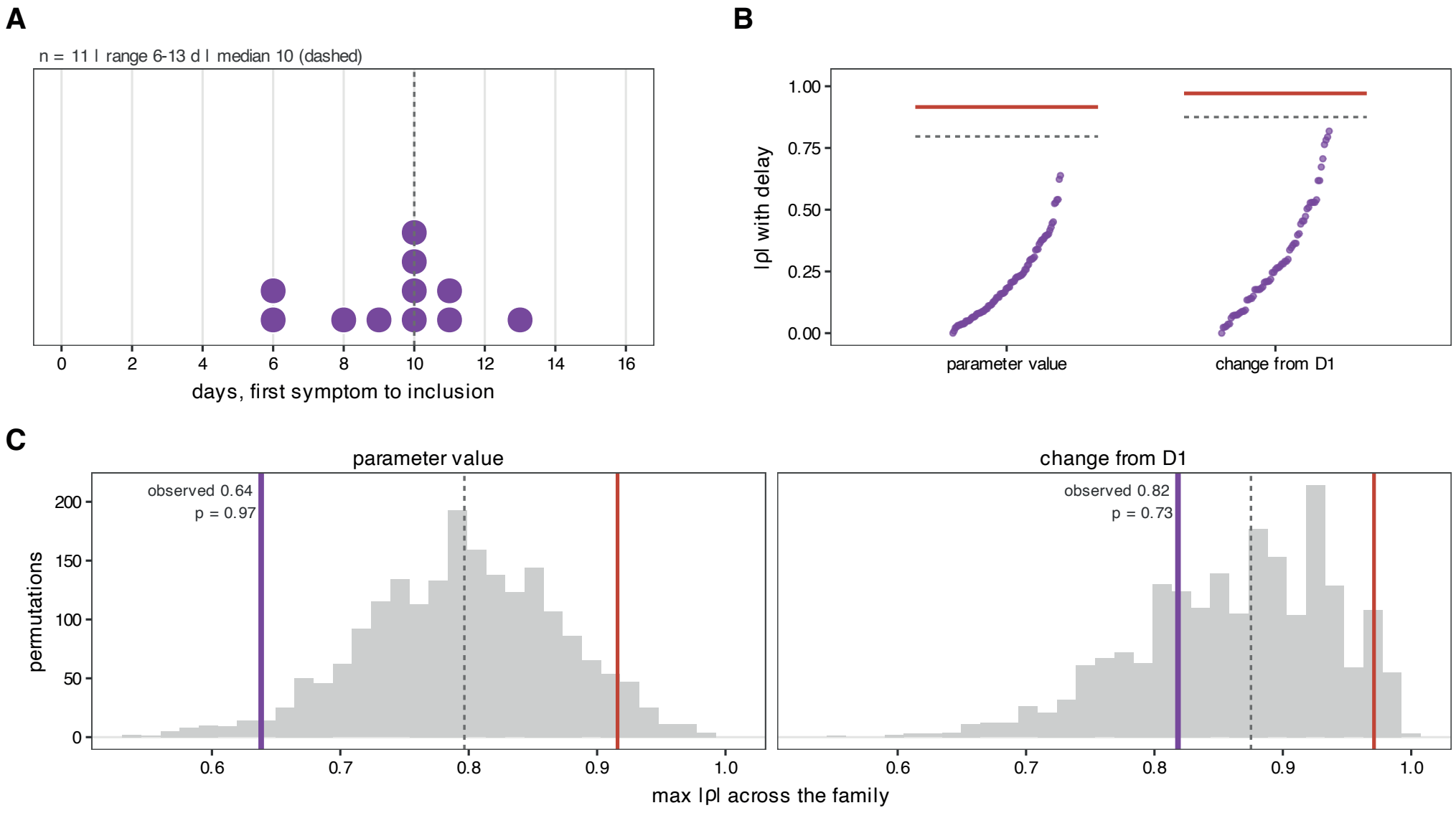

Supplementary Figure 7. B-cell parameters do not vary with the delay from symptom onset to XAV-19 administration. (**A**) Distribution of the delay from first symptom to inclusion across XAV-19–treated patients (n = 11; range 6–13 days; median 10 days, dashed line; 4 patients tied at the modal value). The enrolment window is narrow and entirely late, with no early-treated group. (**B**) Absolute Spearman correlations (|ρ|) between delay and each B-cell parameter, ranked in ascending order within each parameter family (left: parameter value at each timepoint; right: change from D1). The red line marks the 5% significance threshold (95th percentile of the permuted max |ρ|); the dashed line marks the largest correlation expected by chance. Observed correlations fall below the threshold in both families. (**C**) Null distribution of the family-wide max |ρ| from 2,000 permutations of patient delay (grey histogram). The observed max |ρ| (purple line) lies within or below the bulk of the null in both families (parameter value: observed 0.64, p = 0.97; change from D1: observed 0.82, p = 0.73).

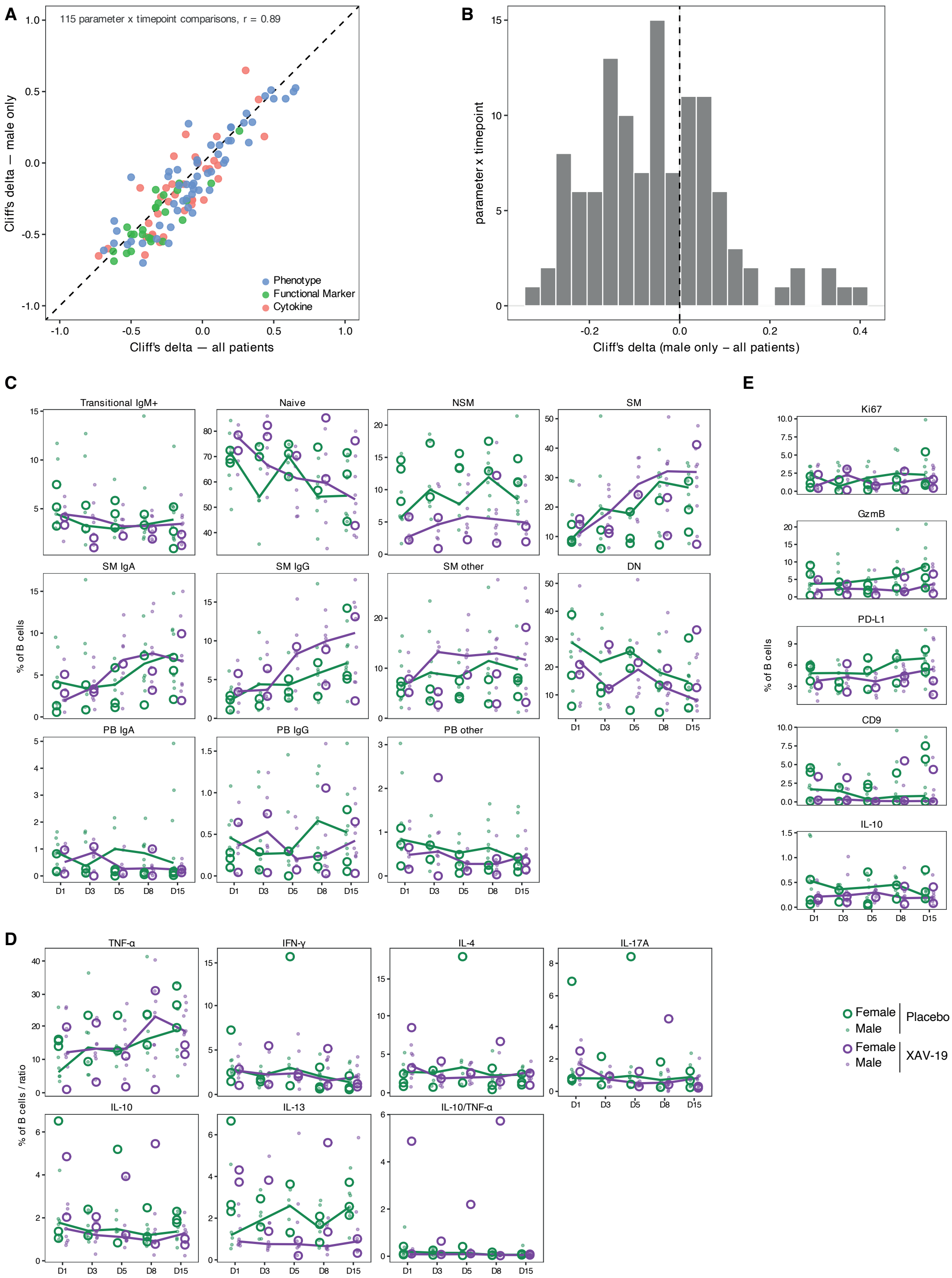

Supplementary Figure 8. B cell remodelling and the XAV-19 treatment effect are robust to cohort sex composition. Sex sensitivity analysis of the flow-cytometry cohort (22 SARS-CoV-2 patients: Placebo n = 11, 8 male/3 female; XAV-19 n = 11, 9 male/2 female) **(A)** Cliff's delta for the XAV-19–versus–placebo comparison estimated in the male-only subset (y-axis)

versus the whole cohort (x-axis) across all 115 parameter × timepoint comparisons, coloured by panel; the dashed line is the identity and points cluster along it (r = 0.89), indicating the treatment contrast is unchanged when women are removed. **(B)** Distribution of the paired differences in Cliff's delta (male only − all patients), centred on zero. **(C–E)** Descriptive overlay of female patients on the male distribution for B cell subsets (**C**), secreted cytokines and the IL-10/TNF-α ratio (**D**), and regulatory-associated functional markers (**E**): male patients are shown as small points with lines connecting the arm-wise male medians, and female patients as open circles; treatment arm is indicated by colour (Placebo, green; XAV-19, purple). No statistics are shown on panels C–E by design (2–4 women per arm). Each point represents one individual.

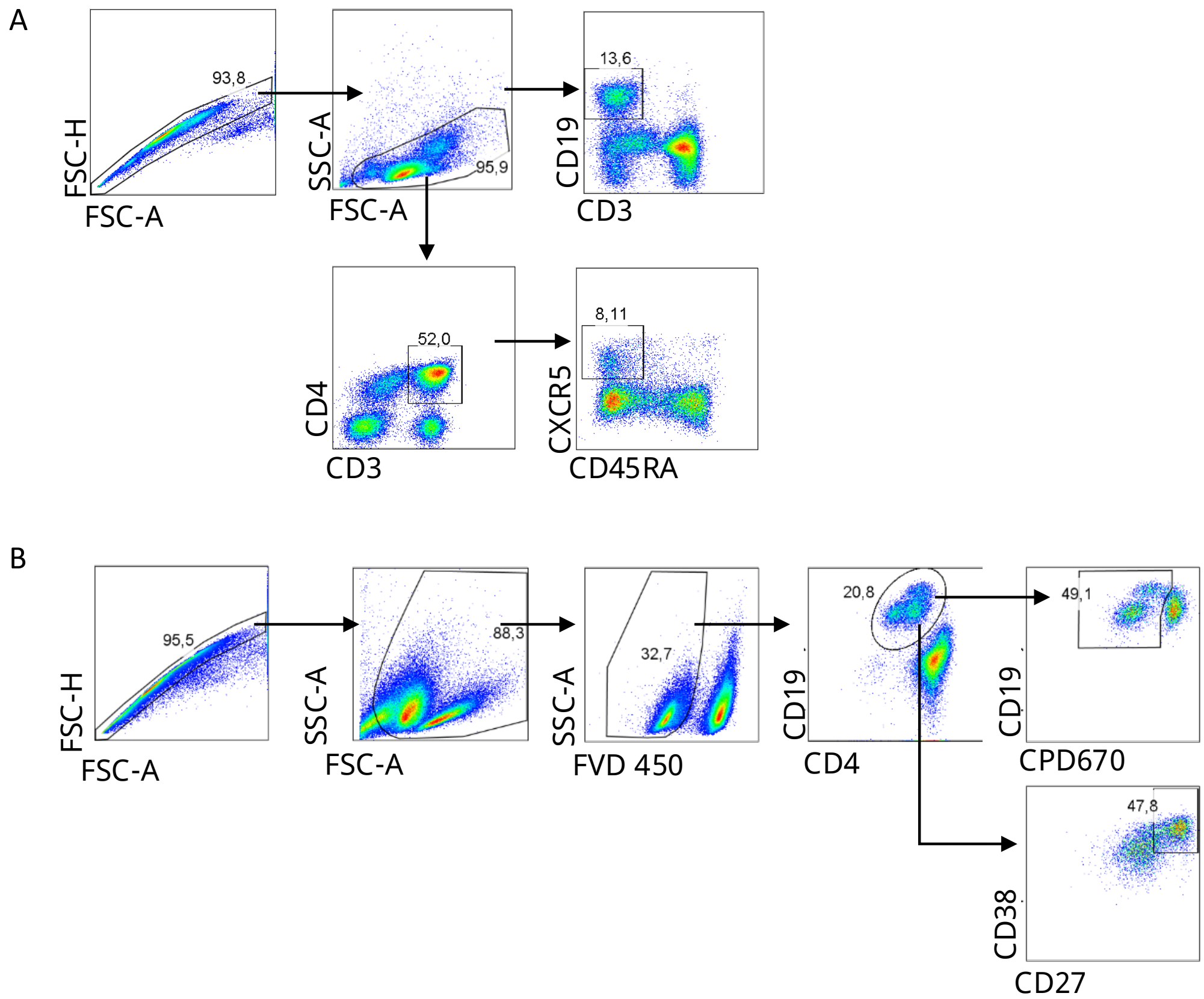

Supplementary Figure 9. Gating strategies used to isolate B cells and follicular helper T cells (Tfh) and to assess B cell proliferation and plasmablast differentiation after coculture. **(A)** Representative gating of B cells ($CD3^{-}CD19^{+}$) and Tfh cells ($CD3^{+}CD4^{+}CD45RA^{-}CXCR5^{+}$). **(B)** Representative gating of proliferating B cells (CPD^{low}) and plasmablasts ($CD27^{hi}CD38^{hi}$) following coculture.

POLYCOR study group*:

Benjamin Gaborit, François Raffi, Maeva Lefebvre, Charlotte Biron, Raphaël Lecomte, Cécile Braudeau, Marie Chauveau, Eric Dailly, Colin Deschanvres, Matthieu Gregoire, Anne-Sophie Lecompte, Eugenie Clapeau, Jérémie Orain, Morgane Le Bras, Alexandre Duval, Isabelle Portier, Clara Mora, Anne-Sophie Boureau, Etienne Seronie-Doutriaux, Agnès Rouaud, Pamela Hublain, Laurence Le Jumeau De Kergaradec, Carole Agasse, Vivien Danielo, Megguy Bernard, Régine Valero, Karine Lacombe, Diane Bollens, Thibault Chiarabini, Nadia Valin, Patrick Ingiliz, Zineb Ouazene, Bénédicte Lefebvre, François Lecardonnel, Christian Tran, Raynald Feliho, Manuela Le Cam, Julie Lamarque, Jean-Luc Lagneau, Cyrielle Letaillandier, Anne Daguenel-Nguyen, Clémentine Mayala-Kanda, Djeneba Fofana, Arianna Fiorentino, Thomas Guimard, Yves Bleher, Jean-Luc Esnault, Dominique Merrien, Blandine Le Claire, Marine Morrier, Delphine Boucher, Romain Lamberet, Clémentine Coudon, Romain Decours, Hélène Durand, Armelle Pegeot, Edwige Migne, Hélène Pelerin, Yannick Poirier, Laurent Hocqueloux, Thierry Prazuck, Barbara De Dieuleveult, Pierre Plocco, Jérôme Guinard, Elisa Demonchy, Eric Cua, Edouard Devaud, Stanislas Harent, Marion Parisey, Céleste Lambert, Elise Gobin, Julien Manson, Pierre Pasquier, Pascale Martres, Patricia Kessedjian, Hikombo Hitoto, Nicolas Crochette, Lucia Perez-Grandiere, Jean-Baptiste Laine, Arnaud Salmon-Rousseau, Guillaume Cosseron, Sophie Blanchi, Florence Ader, Valérie Galvan, Alexia Moulin, Corinne Brochier, Julianne Oddone, Maude Bouscambert-Duchamp, Firouzé Bani-Sadr, Yohan N'guyen, Maxime Hentzien, Cédric Castex, Philippe Benoit, Véronique Brodard, Didier Laureillard, Albert Sotto, Paul Loubet, Aurélie Martin, Régine Doncesco, Julien Mazet, Ian Soulairol, Robin Stephan, François Goehringer, Nathalie Thilly, Michel Prevot, Hélène Jeulin, Jean-Philippe Talarmin, Lydie Khatchatourian, Nadia Saidan, Brice Guerpillon, Pascaline Rameau, Nicolas Cassou, Thomas Briand, Florence Le Gall, Elodie Le Breton, Cédric Joseph, Sandrine Soriot-Thomas, Claire Andrejak, Jean-Philippe Lanoix, Sophie Boddaert, Sandrine

Castelain, Vincent Pestre, Juliette Woessner, Sophie Bayle, Stéphanie Branger, Christine Christides, Philippe Bielefeld, Adèle Lacroix, Roselyne Pillard-Gagliano, Isabelle Trinh, Pierre Lafitte, Guillermo Giordano, Malena Finello, Ignacio Ledesma, Gustavo Martini, Benjamin Delafontaine, Iris Corus, Pierre Baby, Emilie Catherinot, Céline Goyard, Simon Chauveau, Jad Choucair, Beatrice D'urso, Marie Da Silva Costa, Lucie Le Meur, Marc Vasse, Tiffany Pascreau, Eric Farfour, Benjamin Dervieux, C Charlotte Kaeuffer, François Danion, Yves Hansmann, Nicolas Lefebvre, Yvon Ruch, Axel Ursenbach, Catherine Schmidt-Mutter, Muhtadi Suliman, Anne Hutt, Guillaume Becker, Elodie Laugel, Sophie Bayer, Vincent Dubee, Rafael Mahieu, Valérie Daniel, Caroline Lefeuvre, Alexandra Ducancelle, Jean-Charles Gagnard, Abolfzl Mohebbi, Mélanie Dehais, Sophie Raccah, Anne-Lise Pouliquen, Alison Klasen, Emmanuel Forestier, Marie-Christine Carret, Severine Liardot, Jérôme Grosjean, Jean-François Faucher, Josselin Brisset, Anne Cypierre, Hélène Durox, Pauline Pinet, Sophie Ducroix-Roubertou, Claire Genet, Christine Vallejo, Sébastien Hantz, Marie Gousseff, Antoine Merlet, Sébastien Bigot, Marion Girard De Courtilles, Pascal Pouedras, Delphine Lariviere, Renaud Verdon, Sylvie Dargere, Jocelyn Michon, Anna Fournier, Sylvie Brucato, Séverine Gautier, Cécile Valentin, Anne Ricci, Antoine Alix, Flore Lacassin-Beller, Sophie Rousseau, Jérôme Dimet, Anne-Hélène Boivin, Maylis Larregle, Guillaume Rousseau, Ady Assaf, Fanny Vuotto, Karine Faure, Camille Joachim, Laurence Bocket, Kévin Diallo, Jessy Saffore, Isabelle Madeline, Pauline Chabanon, Nathalie Allou, Elisabeth Fernandes, Anne-Sophie Gruliere, Elisabeth Botelho-Nevers, Amandine Gagneux-Brunon, Véronique Ronat, Nadine Casimir, Sylvie Pillet, Frédérique Bertholon, Gilles Pialloux, Marwa Bachir Elrufaai, Ruxandra Calin, Pélagie Thibaut, Fatima Tendjaoui, Julie Fillon, Laurence Morand-Joubert, Marc-Olivier Vareil, Heidi Wille, Philippe Menager, Hugues Cordel, Youssouf Mohammed-Kassim, Vanessa Rathouin, Ségolène Brichler, André Cabie, Mélanie Lehoux, Karine Guitteaud, Karamba Sylla, Bastien Bigeard, Valentine Campana, Isabelle Calmont, Jean-Louis Lamaignere, Marine

Deppenweiller, Christophe Padoin, Marine Thilbault, Laurence Fagour, Fatiha Najioullah, Isabelle Komla-Soukha, Mariam Roncato-Saberan, Martin Martinot, Mahsa Mohseni Zadeh, Simon Gravier, Ciprian Ion, Damien Kayser, Anne Schieber Pachart, Magali Eyriey, Anaïs Henric, Jean Daniel Kaiser, Dominique De Briel, Odile Duvaux, Gwenaëlle Evanno, Pierre-Joseph Royer, Juliette Rousse, Carine Ciron, Elsa Lhériteau, Gaëtane Rouvray, Alan Mougeolle, Auriane Rillet, Sophie Despons, Edwige Mevel, Françoise Shneiker, Régis Josien, Sophie Brouard, Hoa le Mai, Anne Omnès, Ludivine Perrier, Aurélie Le Thuault, Marie-Anne Vibet, Elise Appau-Danquah, Amélie Daniel, Virginie Grybek, David Gueneau, Marion Gautier, Joëlle Martin-Gauthier, Emily Rebouilleau, Joseph Herault, Tanguy Roman, Sorady Plantard, Patrice Chauveau, Alexandra Jobert, Anne Chiffoleau, Stéphanie Thauvin, Isabelle Charreau, Bruno Hoen, Caroline Solas-Chesneau, Astrid Vabret.

Supplementary Table 1. POLYCOR Group author’s list

Variable	Placebo (n=11)	XAV-19 (n=11)	n	p
**Demographics**				
Age, years	53 [49-58] (42-75)	57 [52-62] (41-72)	11/11	0.576
Sex, female	3/11 (27%)	2/11 (18%)	11/11	1.000
BMI, kg/m2	27.8 [26.6-31.1] (22.5-40.0)	28.3 [26.4-31.5] (19.4-34.5)	11/11	0.847
Delay 1st symptom-screening, days	9 [8-10] (4-11)	10 [8-10] (6-13)	11/11	0.547
**Baseline severity**				
SpO2 at rest, %	94 [94-96] (92-96)	93 [92-94] (90-95)	11/11	0.054
NEWS score	6 [4-8] (3-8)	7 [6-9] (4-11)	10/11	0.210
PaO2/FiO2 at D1	261 [206-291] (172-358)	216 [187-240] (157-400)	11/11	0.188
**Baseline biology**				
CRP, mg/L	62.7 [51.7-79.2] (36.3-101.0)	39.4 [26.9-63.8] (15.1-196.7)	8/5	0.354
Ferritin, ug/L	947 [640-1118] (196-1975)	1462 [833-1612] (819-2435)	8/5	0.354
Viral load D1, log	5.0 [4.3-6.7] (0.0-7.2)	5.4 [5.2-5.6] (3.1-5.9)	9/5	0.797
Anti-SARS-CoV-2 serology positive	5/10 (50%)	7/10 (70%)	10/10	0.650
**Co-treatment**				
Corticosteroids at inclusion	9/11 (82%)	9/11 (82%)	11/11	1.000
Corticosteroid delay, days	-1 [-2-0] (-4-3)	-1 [-1-0] (-2-0)	11/9	0.968
**Outcome**				
Ordinal scale D8	4 [2-4] (1-4)	3 [2-4] (1-6)	11/11	0.836
Ordinal scale D15	2 [1-2] (1-3)	2 [2-2] (1-6)	11/11	0.390
PaO2/FiO2 at D8	348 [299-442] (136-457)	410 [290-457] (75-690)	11/11	0.816
PaO2/FiO2 at D15	410 [410-612] (348-762)	410 [336-495] (81-690)	11/11	0.315
Time to discharge, days	10 [8-11] (4-14)	8 [6-10] (4-37)	11/11	0.509
Viral load D8, log	1.1 [0.6-3.7] (0.0-5.2)	0.0 [0.0-1.1] (0.0-5.0)	7/5	0.396
Viral load D15, log	0.0 [0.0-0.6] (0.0-2.8)	0.0 [0.0-0.6] (0.0-1.1)	8/3	1.000
ICU admission by D29	2/11 (18%)	3/11 (27%)	11/11	1.000

Values are median [IQR] (min-max) for continuous variables and n/N (%) for categorical variables. p from a two-sided Mann-Whitney (Wilcoxon rank-sum) test for continuous variables and Fisher exact test for categorical variables.

Supplementary Table 2. Baseline characteristics and clinical outcomes of patients included in the flow cytometry analysis, stratified by treatment group.

Variable	Placebo (n=6)	XAV-19 (n=5)	n	p
**Demographics**				
Age, years	54 [51-58] (48-75)	56 [47-64] (36-74)	6/5	0.931
Sex, female	1/6 (17%)	1/5 (20%)	6/5	1.000
BMI, kg/m2	26.6 [25.5-27.6] (24.7-33.2)	32.3 [25.7-36.0] (25.2-40.6)	6/5	0.360
Delay 1st symptom-screening, days	10 [9-11] (7-11)	7 [6-11] (3-13)	6/5	0.579
**Baseline severity**				
SpO2 at rest, %	94 [93-94] (92-96)	94 [92-94] (91-95)	6/5	0.851
NEWS score	7 [6-8] (4-8)	5 [5-7] (3-10)	5/5	0.598
PaO2/FiO2 at D1	233 [199-257] (172-300)	182 [182-282] (172-350)	6/5	0.927
**Baseline biology**				
CRP, mg/L	72.9 [42.7-103.2] (36.3-110.0)	29.1 (14.4-43.7)	4/2	0.267
Ferritin, ug/L	732 [456-956] (267-988)	577 (220-934)	4/2	0.533
Viral load D1, log	4.4 [3.9-5.0] (2.7-7.0)	2.2 (1.1-3.3)	4/2	0.267
Anti-SARS-CoV-2 serology positive	3/6 (50%)	4/4 (100%)	6/4	0.200
**Co-treatment**				
Corticosteroids at inclusion	5/6 (83%)	5/5 (100%)	6/5	1.000
Corticosteroid delay, days	-1 [-1--0] (-2-1)	-2 [-2--1] (-2-0)	6/5	0.249
**Outcome**				
Ordinal scale D8	3 [1-4] (1-4)	3 [3-4] (1-4)	6/5	0.923
Ordinal scale D15	2 [1-2] (1-3)	2 [2-2] (1-2)	6/5	0.682
PaO2/FiO2 at D8	353 [284-401] (136-457)	310 [304-410] (276-533)	6/5	0.855
PaO2/FiO2 at D15	410 [410-445] (348-533)	410 [410-457] (410-533)	6/5	0.689
Time to discharge, days	9 [6-11] (4-14)	5 [4-10] (4-11)	6/5	0.351
Viral load D8, log	3.9 [2.2-5.2] (1.1-5.4)	0.0	4/1	0.400
Viral load D15, log	0.0 [0.0-0.0] (0.0-2.8)	0.0	5/1	1.000
ICU admission by D29	2/6 (33%)	0/5 (0%)	6/5	0.455

Values are median [IQR] (min-max) for continuous variables and n/N (%) for categorical variables. p from a two-sided Mann-Whitney (Wilcoxon rank-sum) test for continuous variables and Fisher exact test for categorical variables.

Supplementary Table 3. Baseline characteristics and clinical outcomes of patients included in the scRNAseq analysis, stratified by treatment group.

Variable	Placebo (n=12)	XAV-19 (n=17)	n	p
**Demographics**				
Age, years	54 [49-60] (43-75)	56 [47-58] (36-83)	12/17	0.825
Sex, female	2/12 (17%)	4/17 (24%)	12/17	1.000
BMI, kg/m2	27.0 [25.1-31.8] (22.5-40.0)	31.2 [27.7-33.6] (23.2-40.6)	12/17	0.341
Delay 1st symptom-screening, days	10 [9-11] (7-11)	9 [6-10] (3-13)	12/17	0.185
**Baseline severity**				
SpO2 at rest, %	94 [93-96] (92-96)	93 [92-94] (90-95)	12/17	0.032
NEWS score	5 [4-7] (3-8)	8 [5-10] (3-12)	11/17	0.038
PaO2/FiO2 at D1	254 [222-302] (172-358)	232 [192-261] (172-350)	12/17	0.329
**Baseline biology**				
CRP, mg/L	78.2 [68.2-101.0] (36.3-129.6)	63.8 [26.9-127.8] (14.4-196.7)	9/9	0.436
Ferritin, ug/L	947 [513-1502] (267-1975)	859 [819-1272] (220-2435)	8/9	0.815
Viral load D1, log	4.4 [4.1-6.6] (0.0-6.7)	4.2 [3.2-5.4] (1.1-7.4)	9/6	0.955
Anti-SARS-CoV-2 serology positive	5/10 (50%)	12/16 (75%)	10/16	0.234
**Co-treatment**				
Corticosteroids at inclusion	10/12 (83%)	16/17 (94%)	12/17	0.553
Corticosteroid delay, days	-1 [-1-0] (-4-3)	-1 [-2-0] (-2-0)	12/16	0.382
**Outcome**				
Ordinal scale D8	3 [2-4] (1-4)	3 [2-4] (1-4)	12/17	0.981
Ordinal scale D15	2 [2-2] (1-3)	2 [1-2] (1-4)	12/17	0.732
PaO2/FiO2 at D8	393 [309-457] (136-457)	329 [304-457] (246-690)	12/17	0.893
PaO2/FiO2 at D15	410 [410-476] (348-690)	457 [376-457] (295-690)	12/17	0.964
Time to discharge, days	8 [7-11] (5-14)	7 [5-10] (4-23)	12/17	0.265
Viral load D8, log	4.5 [1.9-5.1] (0.0-5.4)	0.0 [0.0-1.8] (0.0-4.1)	6/8	0.037
Viral load D15, log	0.0 [0.0-0.6] (0.0-1.1)	0.0 [0.0-0.0] (0.0-3.9)	7/6	0.846
ICU admission by D29	2/12 (17%)	3/17 (18%)	12/17	1.000

Values are median [IQR] (min-max) for continuous variables and n/N (%) for categorical variables. p from a two-sided Mann-Whitney (Wilcoxon rank-sum) test for continuous variables and Fisher exact test for categorical variables.

Supplementary Table 4. Baseline characteristics and clinical outcomes of patients included in the B/TFH analysis, stratified by treatment group.

Target	Fluorochrome	Clone Name	Provider	Assay[1]
**CD19**	BUV805	SJ25C1	BD Biosciences	1
**CD19**	PerCP/Cyanine 5.5	HIB19	Biolegend	2, 3, 4
**CD25**	PE/Fire 700	M-A251	Biolegend	1
**CD123**	Alexa Fluor 647	9F5	BD Biosciences	1
**IgD**	Alexa Fluor 700	IA6-2	Biolegend	1
**IFNg**	APC	B27	BD Biosciences	2
**CD27**	APC	M-T271	Biolegend	1
**CD27**	PE-Cy7	B49205	Beckman Coulter	4
**CD56**	APC/Fire 750	5.1H11	Biolegend	1
**CCR7**	APC/Fire 810	G043H7	Biolegend	2
**CD38**	APC/Fire 810	HIT2	Biolegend	1
**CD38**	PE	HIT2	BD Biosciences	4
**CD141**	BB515	1A4	BD Biosciences	1
**KI-67**	BUV395	B56	BD Biosciences	1
**CD16**	BUV496	3G8	BD Biosciences	1
**CD45RA**	BUV496	HI100	BD Biosciences	2
**CD45RA**	Pe	HI100	BD Biosciences	3
**CD158b**	BUV563	CH-L	BD Biosciences	1
**CXCR5**	BUV563	RF8B2	BD Biosciences	2
**CXCR5**	AlexaFluor 488	RF8B2	BD Biosciences	3
**CD159C**	BUV615	134591	BD Biosciences	1
**CD11c**	BUV661	B-ly6	BD Biosciences	1
**CD138**	BUV737	MI15	BD Biosciences	1
**CD56**	BUV737	NCAM16.2	BD Biosciences	2
**CD3**	BUV805	OKT3	BD Biosciences	2
**CD3**	PE-Cy5	HIT3a	BD Biosciences	1
**CD3**	APC	SP34-2	BD Biosciences	3
**IL-13**	BV421	JES10-5A2	BD Biosciences	2
**IL-10**	BV421	JES3-9D7	Biolegend	1
**CD9**	BV510	M-L13	BD Biosciences	1
**IgM**	BV570	MHM-88	Biolegend	1
**IgG**	BV605	G18-145	BD Biosciences	1
**CD159a**	BV650	131411	BD Biosciences	1
**CD158a**	BV711	HP-3E4	BD Biosciences	1
**IL-10**	BV711	JES3-9D7	BD Biosciences	2
**CD158e1**	BV750	DX9	BD Biosciences	1
**PD-1**	BV785	EH12.2H7	Biolegend	1
**CD4**	CF568	SK3	Cytek	2
**CD4**	V450	RPA-T4	BD Biosciences	3
**CD4**	FITC	RPA-T4	BD Biosciences	4

**IgA**	FITC	polyclonal	Jackson ImmunoResearch	1
**TNFa**	FITC	cA2	Miltenyi	2
**CD16**	Pacific Orange	3G8	Thermo Fischer Scientific	2
**CD20**	Pacific Orange	HI47	Thermo Fischer Scientific	1
**Gzm B**	PE	GB11	BD Biosciences	1
**IL-17a**	PE	eBio64DEC17	eBioscience	2
**IL4**	PE-Cy7	8D4-8	BD Biosciences	2
**CD274 (PD-L1)**	PE-Cy7	MIH1	BD Biosciences	1
**CD24**	PE/Dazzle 594	ML5	Biolegend	1
**HLA-DR**	PE/Fire 810	L243	Biolegend	1
**CD45**	PerCP	2D1	Biolegend	1,2
**TCRgd**	PerCP-Vio700	REA591	Miltenyi	2
**CD11b**	PerCP/Cyanine 5.5	ICRF44	Biolegend	1
**CD14**	Spark Blue 550	63D3	Biolegend	1
**CD8**	Spark Blue 550	SK1	Biolegend	2

Spectral cytometry – B cell Panel (1), Spectral cytometry –Functional Panel (2), Conventional cytometry – Cell sorting (3), Conventional cytometry – B cell proliferation and differentiation (4)

Supplementary Table 5. Listing of antibodies

Reagent or Resource	Provider
PMA	Sigma
Calcium Ionophore	Sigma
BrefeldineA	Sigma
e-Biosciences CytoFix/Perm kit	Thermofisher
CPD eFluor 670	Thermofisher
FVD eFluor 450	Thermofisher
CTL Wash supplement 10X	Immunospot
Benzonase	Sigma
Brillant Stain Buffer Plus	BD
Live/dead Fixable Blue for UV excitation	ThermoFischer

CPD: cell proliferation dye; FVD: fixable viability dye

Supplementary Table 6. Listing of reagents
